# Supplementary material for: An ALYREF-MYCN coactivator complex drives neuroblastoma tumorigenesis through effects on USP3 and MYCN stability
Source: Nat Commun. 2021 Mar 25;12:1881. doi: 10.1038/s41467-021-22143-x (PMC7994381; doi:10.1038/s41467-021-22143-x)
Supplement: Supplementary file 1 — Supplementary Information [file 41467_2021_22143_MOESM1_ESM.pdf]

Supplementary Information

An ALYREF-MYCN coactivator complex drives neuroblastoma tumorigenesis through effects on USP3 and MYCN stability

Z Nagy et al.

Supplementary Figure 1

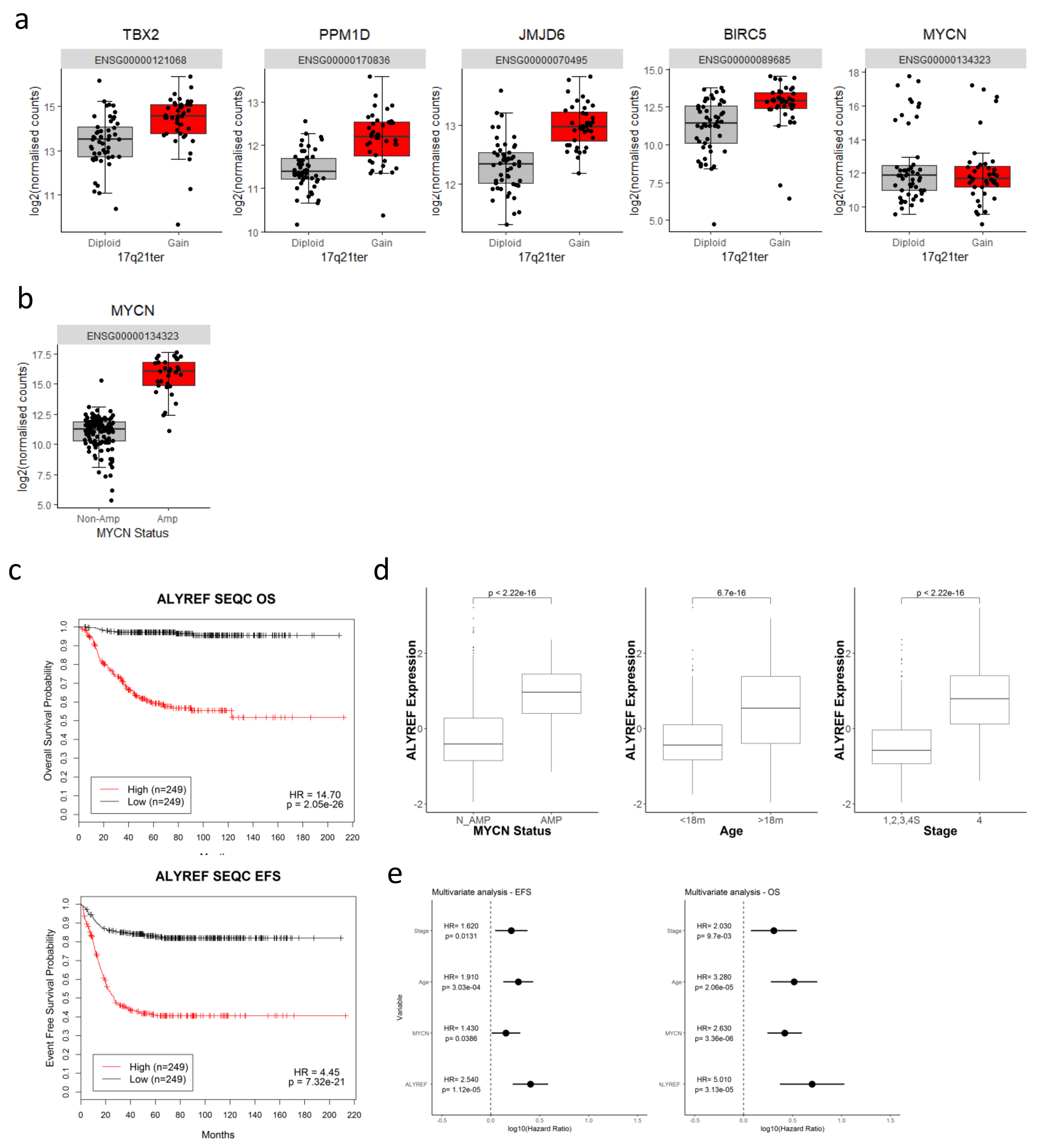

Supplementary Figure 1 (cont'd)

f

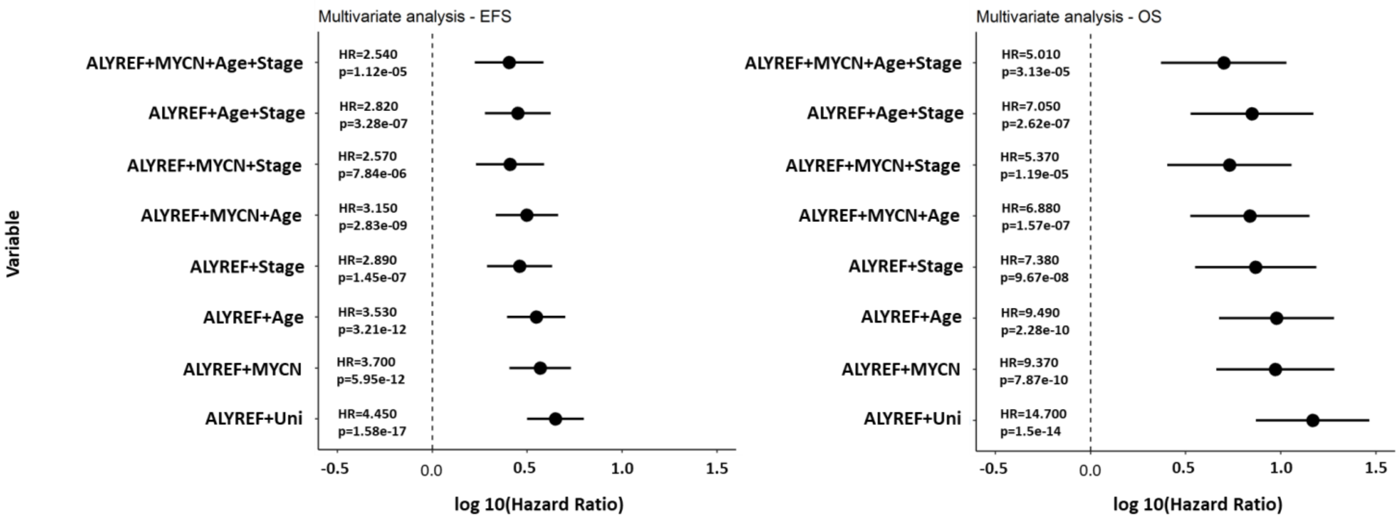

g

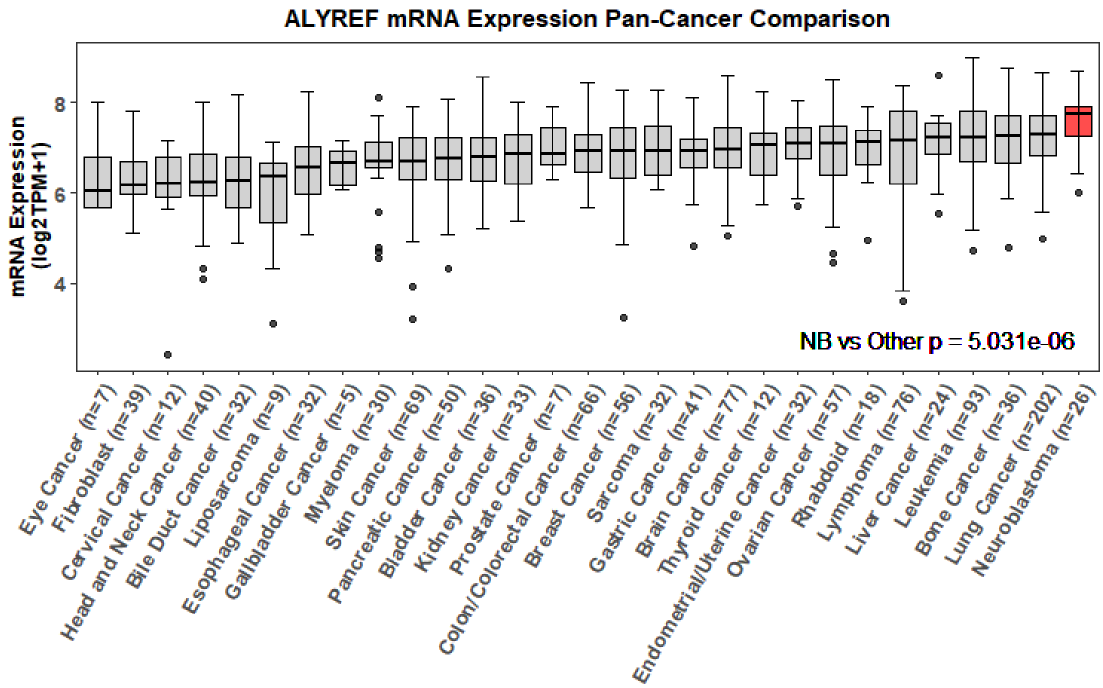

h

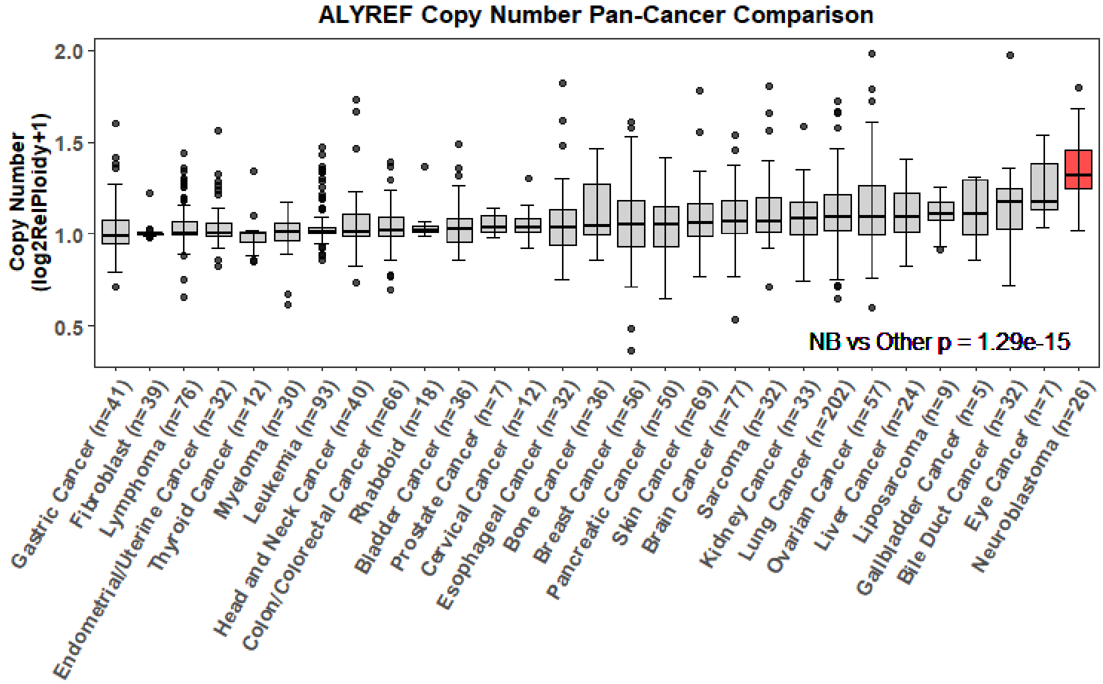

Supplementary Figure 1 (cont'd)

i

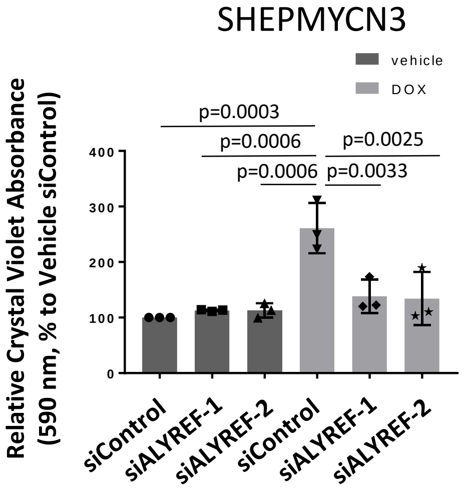

j

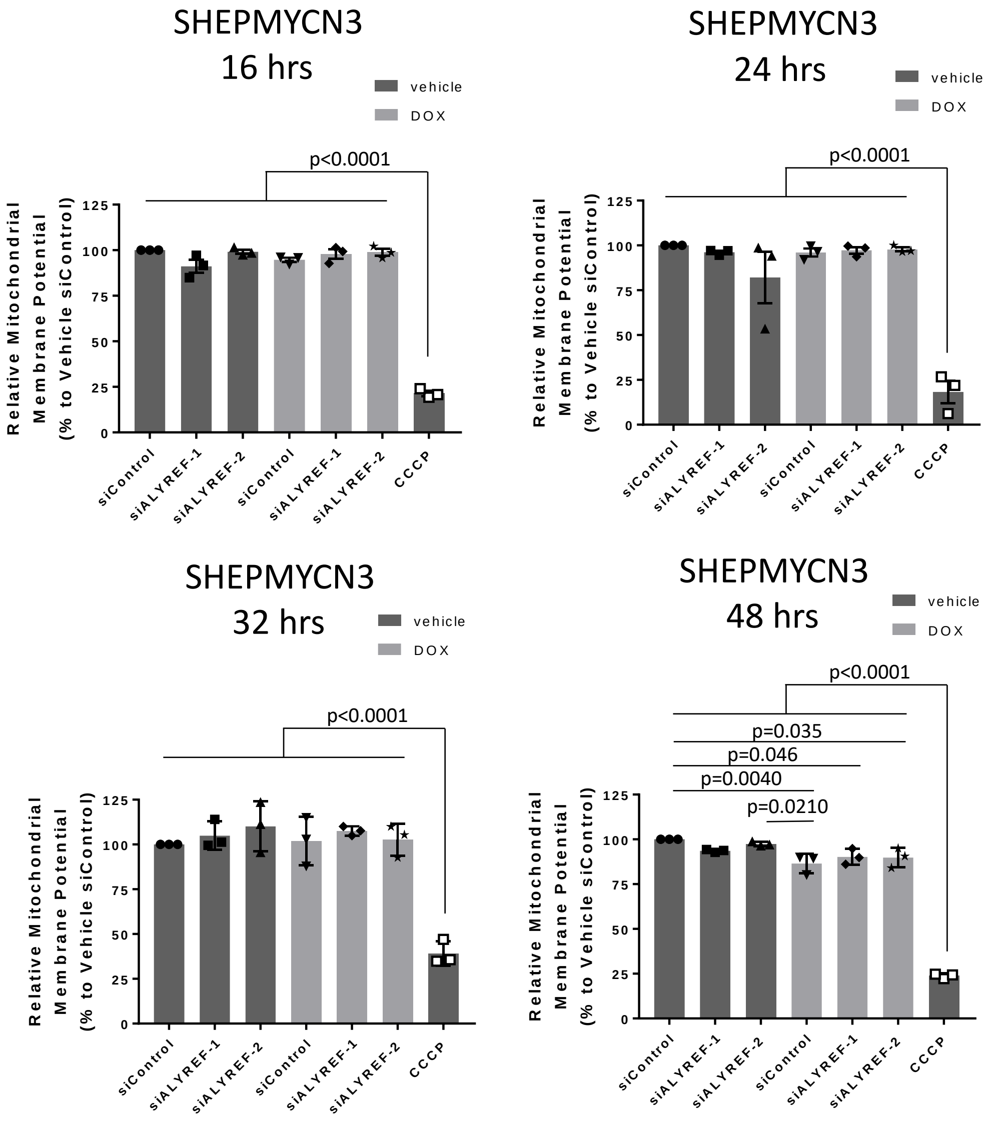

Supplementary Figure 1 (cont'd)

k

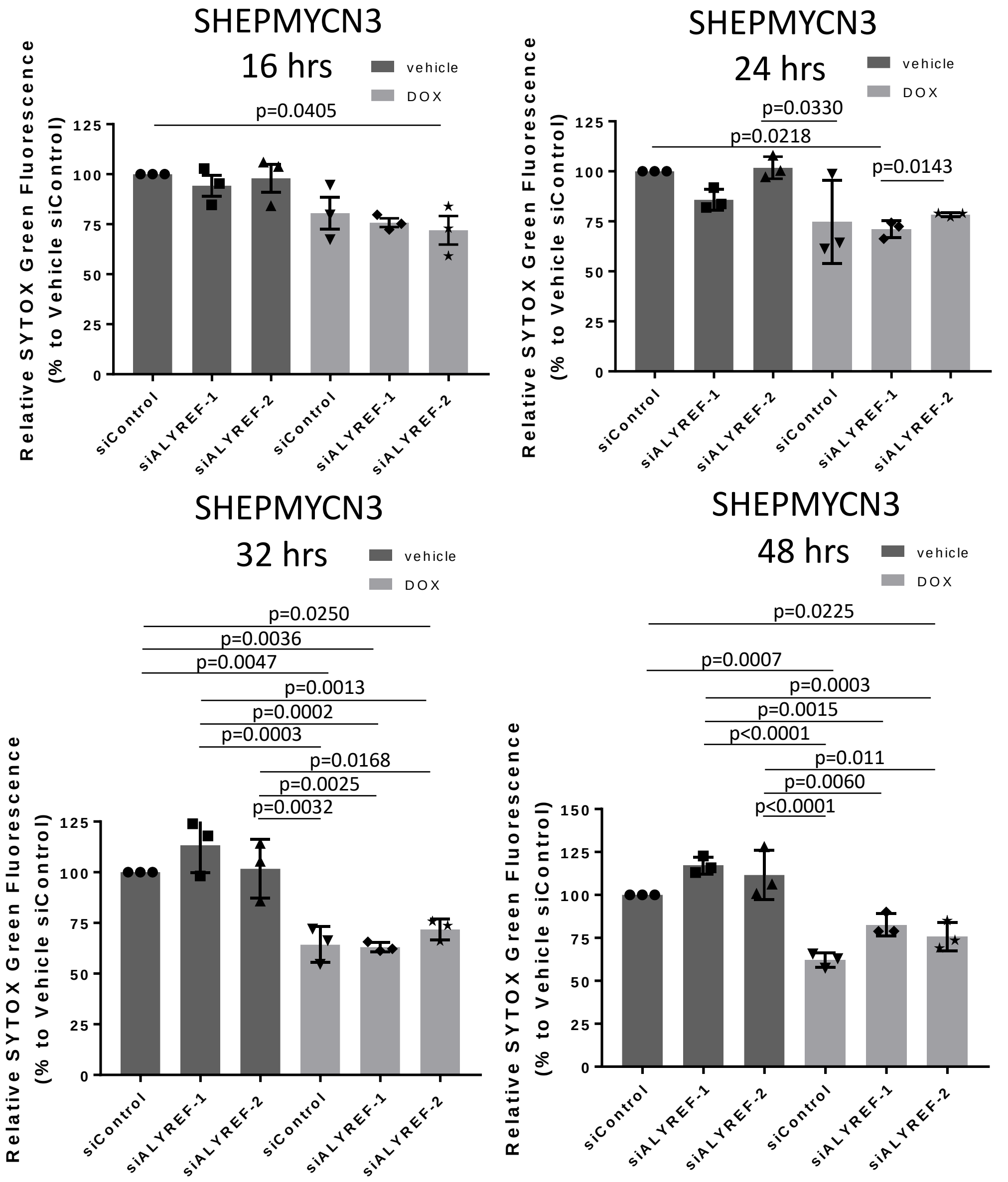

l

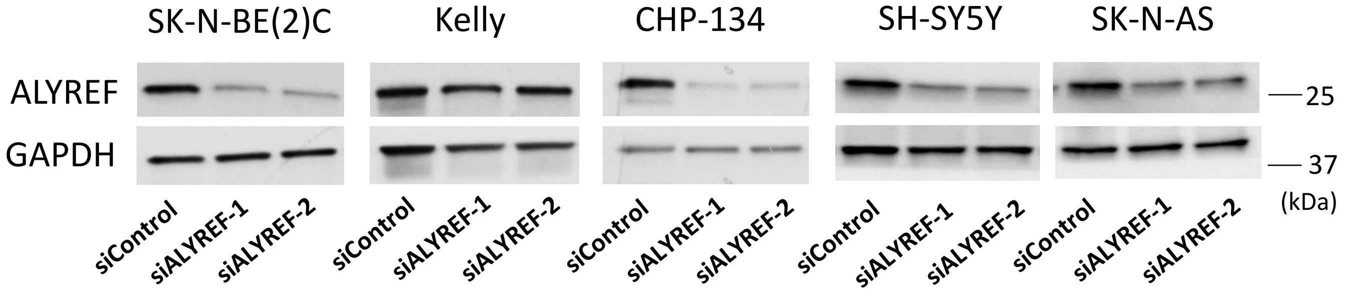

m

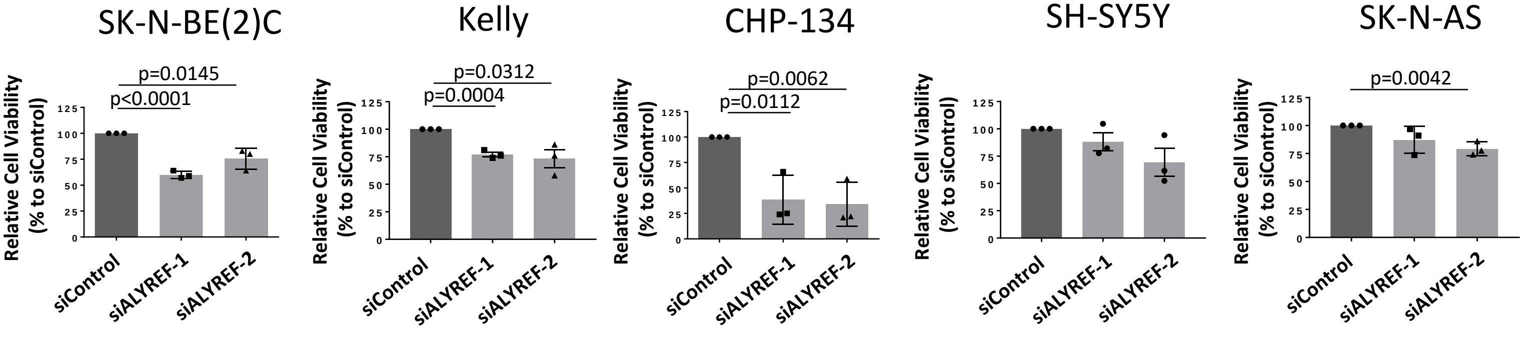

Supplementary Figure 1 (cont'd)

Supplementary Figure 1. High ALYREF expression associates with 17q21-ter gain, *MYCN*-amplification and poor patient prognosis in neuroblastoma, related to Fig. 1

(a) Boxplots displaying *TBX2, PPM1D, JMJD6, BIRC5* and *MYCN* expression between diploid (n=50) and 17q21-ter gain (n=40) patients. (b) Boxplot displaying *MYCN* expression between *MYCN*-non-amplified (n=121) and *MYCN*-amplified (n=33) patients. (c) Kaplan-Meier survival curves showing overall and event-free survival probability of patients in the SEQC neuroblastoma cohort (n=498) when dichotomised by median *ALYREF* expression (RNA-Seq). Hazard Ratio's (HR) and two-sided log-rank p-values are presented from a univariate CoxPH model. (d) *ALYREF* expression in tumors dichotomised by *MYCN*-amplification status (non-amplified vs. amplified), and stage (advanced stage: 3, 4 vs others stage: 1, 2, 4S), and age at diagnosis (<18 months vs > 18 months), n = 498. Two-sided unpaired Student's t-tests were performed to derive p-values. (e) Multivariate overall and event-free survival analysis dichotomised by stage of disease, age of patient at diagnosis, *MYCN* expression, and *ALYREF* expression using CoxPH modelling. The p-values were obtained using two-sided log-rank tests. (f) Multivariate overall and event-free survival analysis further dichotomised by *ALYREF* expression, stage of disease, age of patient at diagnosis and *MYCN* expression using cox regression modelling. The p-values were obtained using two-sided log-rank tests. (g) *ALYREF* gene expression in transcripts per million (TPM) across 29 cancer types using the Cancer Cell Line Encyclopedia (CCLE) database, p-value is form a two-sample t-test between neuroblastoma cell lines and an aggregate of all other cell lines, samples are ranked by median expression. (h) ALYREF copy number expressed as relative to ploidy (2n) across 29 cancer types using the Cancer Cell Line Encyclopedia (CCLE) database, p-value is form a two-sample t-test between neuroblastoma cell lines and an aggregate of all other cell lines, samples are ranked by median copy number. (i) Quantification of colony forming assay (n=3 per group) for doxycycline induced (DOX) vs non-induced (Vehicle) SHEPMYCN3 neuroblastoma cells transfected with ALYREF siRNA-1, ALYREF siRNA-2 or Control siRNA (siControl) based on crystal violet absorbance (590nm). Differences in colony formation were compared to the vehicle treated control siRNA (Vehicle siControl). Two-sided one-way ANOVA was performed to derive p-values. Doxycycline induced (DOX) vs non-induced (Vehicle) SHEPMYCN3 neuroblastoma cells transfected with ALYREF siRNA-1, ALYREF siRNA-2 or Control siRNA (siControl) subjected to (j) MitoProbe DILC1(5) (n=3 per group) and (k) SYTOX Green measurements (n=3 per group) at 16-48 hours. CCCP (carbonyl cyanide m-chlorophenyl hydrazone) served as positive control. Differences in fluorescence signals were normalized to the vehicle treated control siRNA (Vehicle siControl). Two-sided one-way ANOVA was performed to derive p-values. (l) Immunoblots of ALYREF expression in neuroblastoma cells (SK-N-BE(2)C, Kelly, CHP-134, SH-SY5Y and SK-N-AS) following siRNA-mediated ALYREF knockdown for 48 hours. (m) Neuroblastoma cells (SK-N-BE(2)C, Kelly, CHP-134, SH-SY5Y, SK-N-AS) expressing ALYREF siRNAs or control siRNAs subjected to cell viability measurements (n=3 per group) at 72 (SH-SY5Y, SK-N-AS) and 96 (SK-N-BE(2)C, Kelly, CHP-134) hours. Differences in cell growth were compared to Control siRNA transfected cells. Two-sided unpaired Student's t-tests were performed to derive p-values. Respective p values (p) on all figures are displayed. Comparisons were not significant unless otherwise noted. Data are shown as mean ± s.e.m. (error bars) and representative of three independent experiments in i-k and m. Data is representative of three independent experiments with similar results in l. The dot (measure of centre for the error bars) in each row represents the mean hazard ratio, while the error bars represent the 95% confidence intervals for the presented mean hazard ratio in e and f. For each boxplot, the line in the middle of the box represents the median expression value, and the upper/lower bounds of the boxes represent the interquartile range of all expression values (3rd quartile and 1st quartile respectively) in a, b, d, g and h. The whiskers represent 1.5x the interquartile range of all expression values, from the upper/lower bounds of the box in a, b, d, g and h.

Supplementary Figure 2

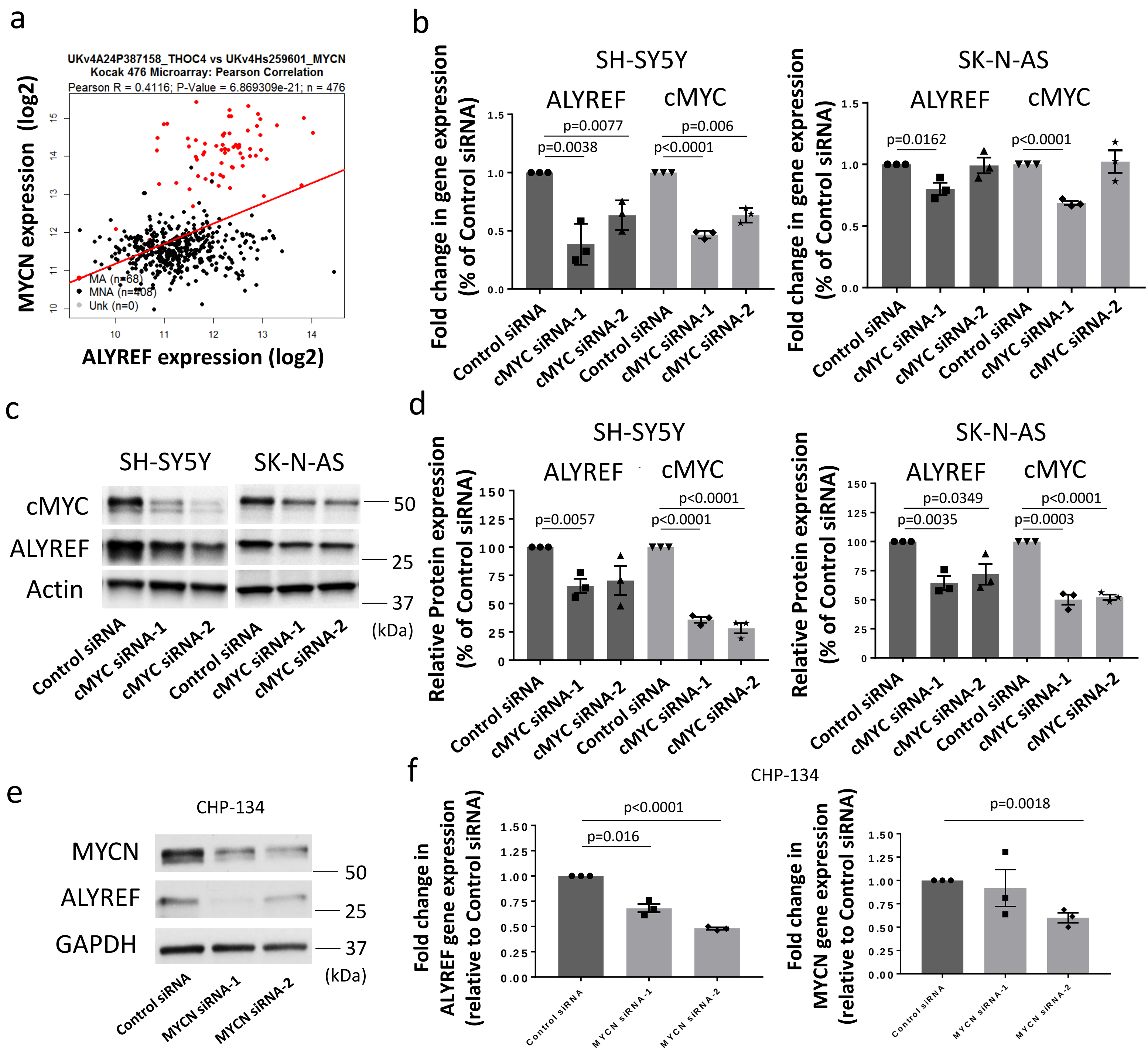

Supplementary Figure 2. MYCN directly upregulates ALYREF transcription, related to Fig. 2

(a) Scatter plot, with a linear regression fit for 476 neuroblastoma patients from the Kocak cohort for *MYCN* vs *ALYREF* (also known as *THOC4*) gene expression (log2 expression). Pearson correlation coefficient (r) and respective p-value (p) are also displayed. Two-sided Pearson's correlation test was used to derive p values. (b) Q-PCR analysis (n=3 per group) of *ALYREF* and *cMYC* expression in SH-SY5Y and SK-N-AS cells following siRNA-mediated cMYC knockdown. Two-sided unpaired Student's t-tests were performed to derive p values. (c) Immunoblot and (d) densitometry analysis (n=3 per group) of ALYREF and cMYC protein expression in SH-SY5Y and SK-N-AS cells following siRNA-mediated cMYC knockdown. Differences expression were compared to Control siRNA transfected cells. Two-sided unpaired Student's t-tests were performed to derive p-values. Immunoblot (e) and Q-PCR (f) analysis (n=3 per group) of ALYREF and MYCN expression in CHP-134 cells following siRNA-mediated MYCN knockdown. Differences in expression were compared to Control siRNA transfected cells. Two-sided unpaired Student's t-tests were performed to derive p-values. Respective p values (p) on all figures are displayed. Comparisons were not significant unless otherwise noted. Data is representative of three independent experiments with similar results in c and e. Data are shown as mean ± s.e.m. (error bars) and representative of three independent experiments in b, d and f.

Supplementary Figure 3

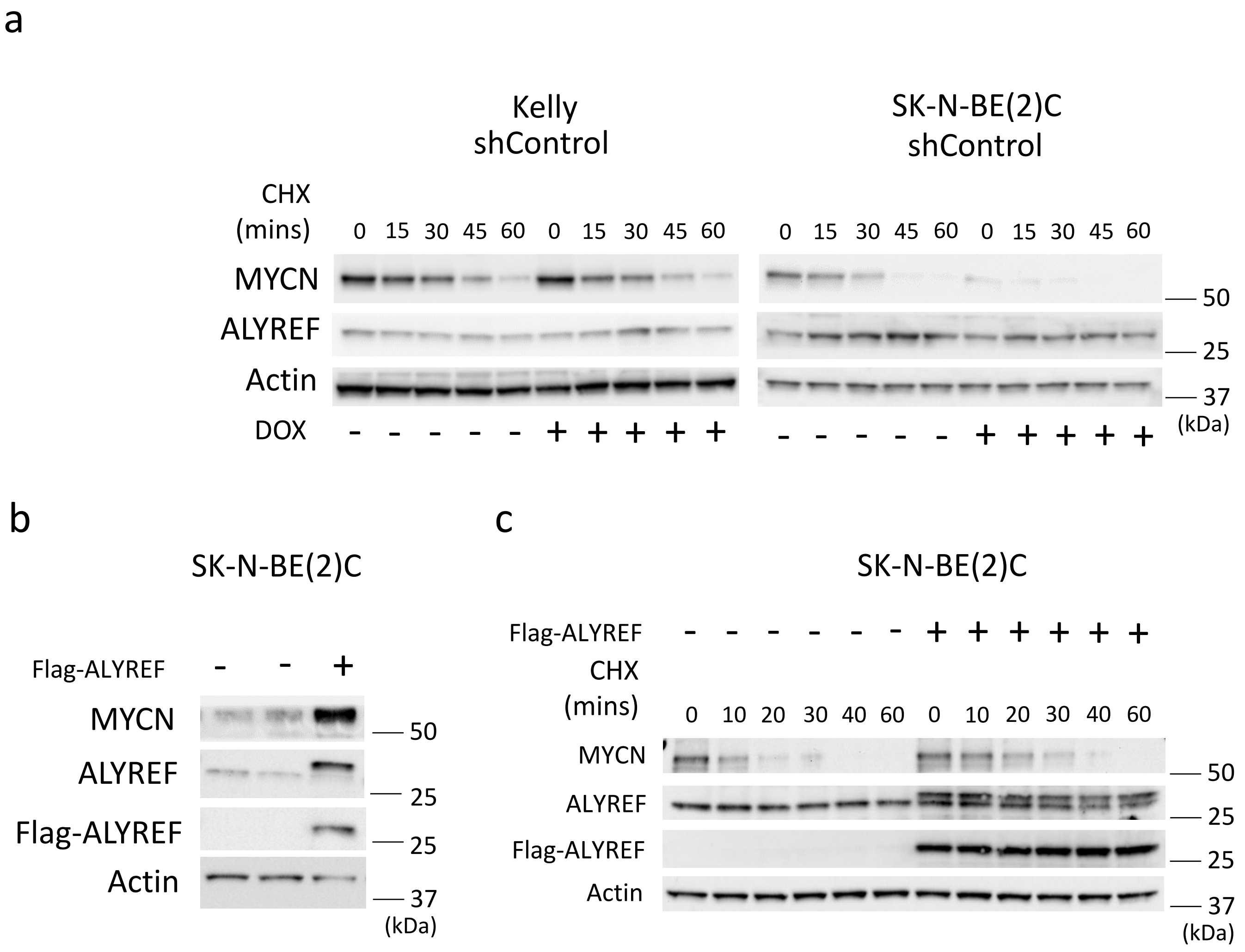

Supplementary Figure 3. ALYREF regulates MYCN stability in a forward feedback expression loop, related to Fig. 3

(a) Kelly and SK-N-BE(2)C cells expressing Control shRNA (shControl) were treated with doxycycline (DOX; 2 µg/ml) for 72 hours, followed by treatment with 100ug/ml cycloheximide (CHX) for 0, 15, 30, 45, or 60 mins. Protein was extracted and subjected to immunoblot analysis of MYCN and ALYREF expression. (b) Immunoblot of SK-N-BE(2)C cells expressing either Flag-ALYREF or empty vector control ("-") at 48 hours. (c) SK-N-BE(2)C cells expressing either Flag-ALYREF or empty vector control ("-") for 48 hours were treated with 100ug/ml cycloheximide (CHX) for 0 , 15, 30, 45, or 60 mins. Protein was extracted and subjected to immunoblot analysis of MYCN and ALYREF expression. Data is representative of three independent experiments with similar results in a-c.

Supplementary Figure 4

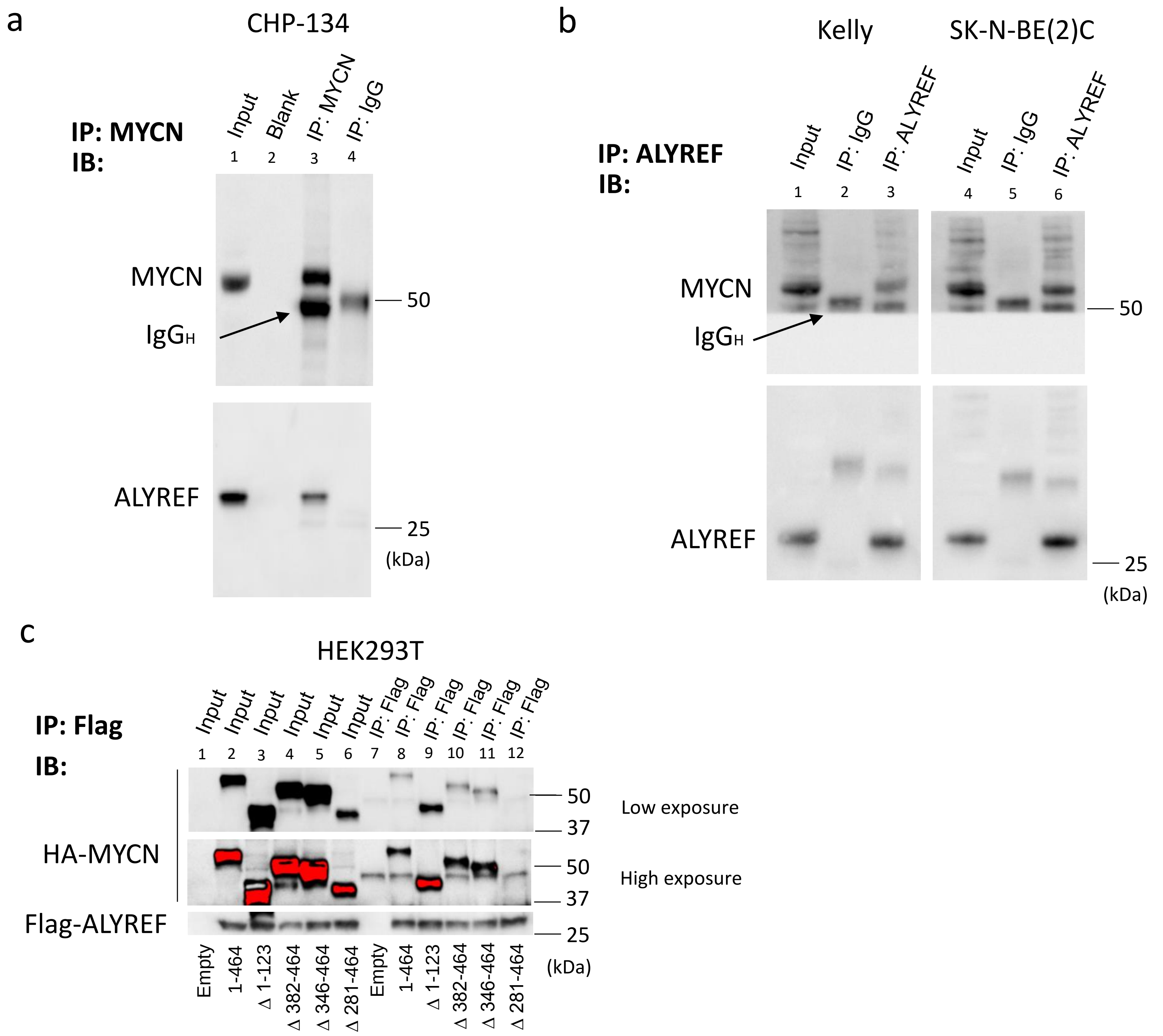

Supplementary Figure 4. ALYREF forms a protein complex with MYCN in the nucleus, related to Fig. 4
(a) Immunoblot analysis for endogenous ALYREF after immunoprecipitation of endogenous MYCN from *MYCN*-amplified CHP-134 cells. 5% of the cell lysate was loaded for input. (b) Immunoblot analysis for endogenous MYCN after immunoprecipitation of endogenous ALYREF from *MYCN*-amplified Kelly and SK-N-BE(2)C cells. 5% of the cell lysate was loaded for input. (c) Immunoblot analysis using low- and high exposure time for the indicated ectopically overexpressed HA-MYCN deletion mutants and Flag-ALYREF from HEK293T cells after immunoprecipitation of Flag-ALYREF. 5% of the cell lysate was loaded for input. Empty indicates HEK293T cells that were transfected with an empty vector. Data is representative of three independent experiments with similar results in a-c.

Supplementary Figure 5

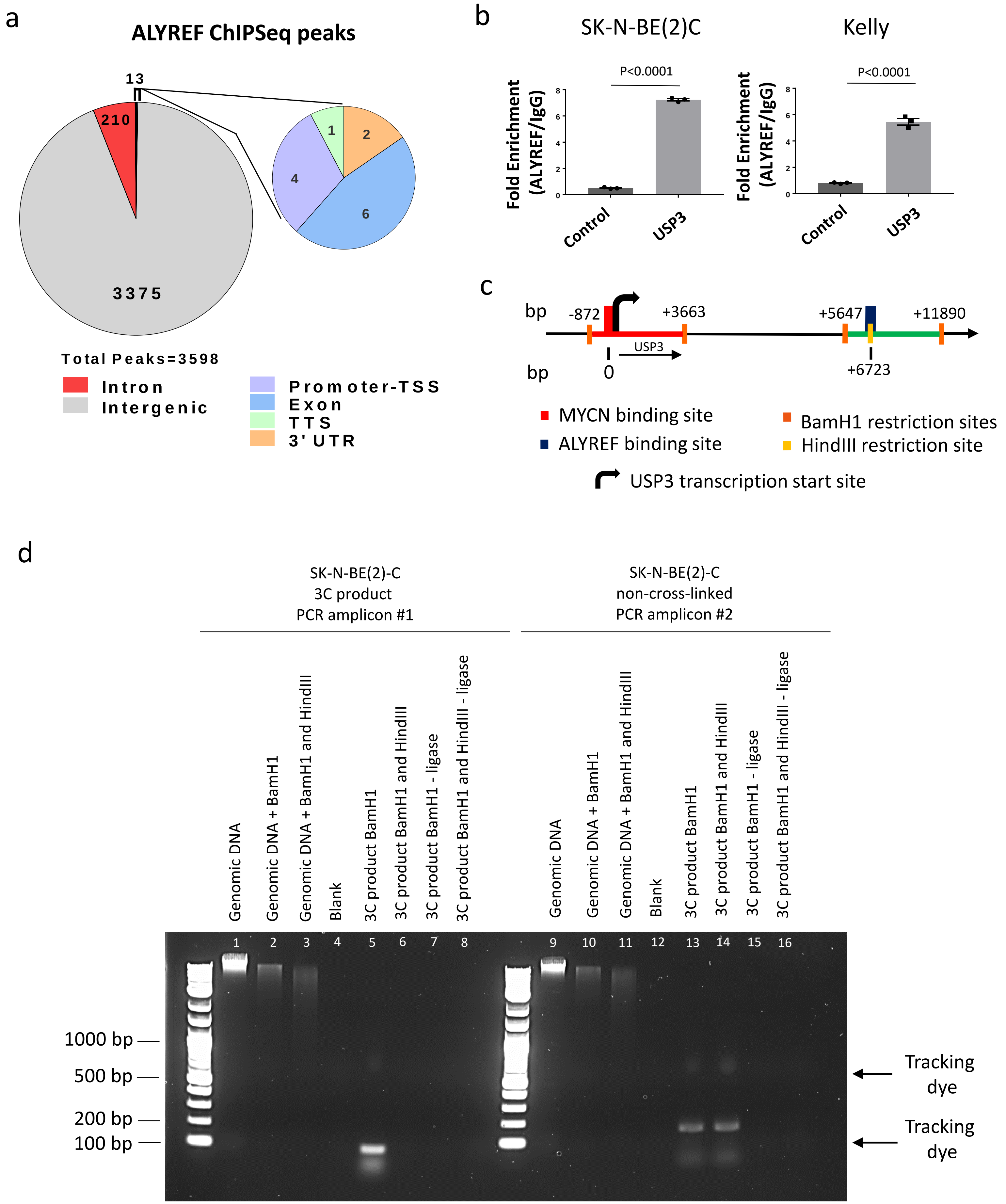

Supplementary Figure 5 (cont'd)

e

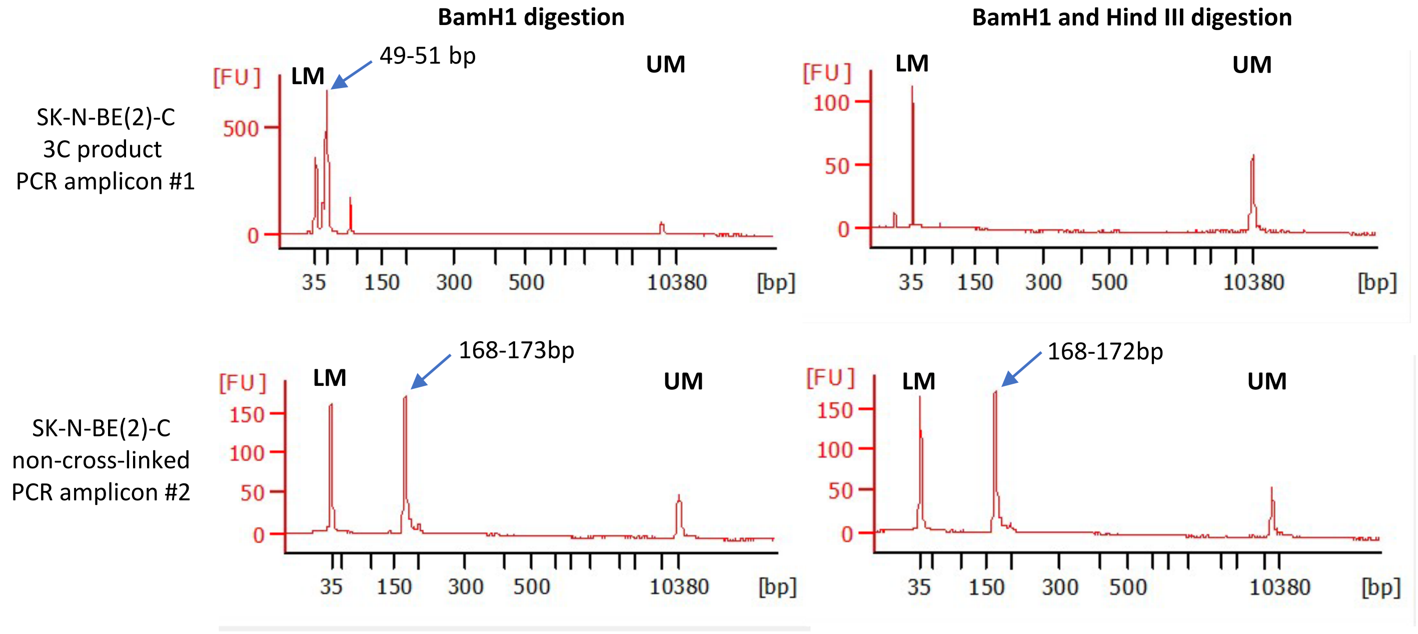

f

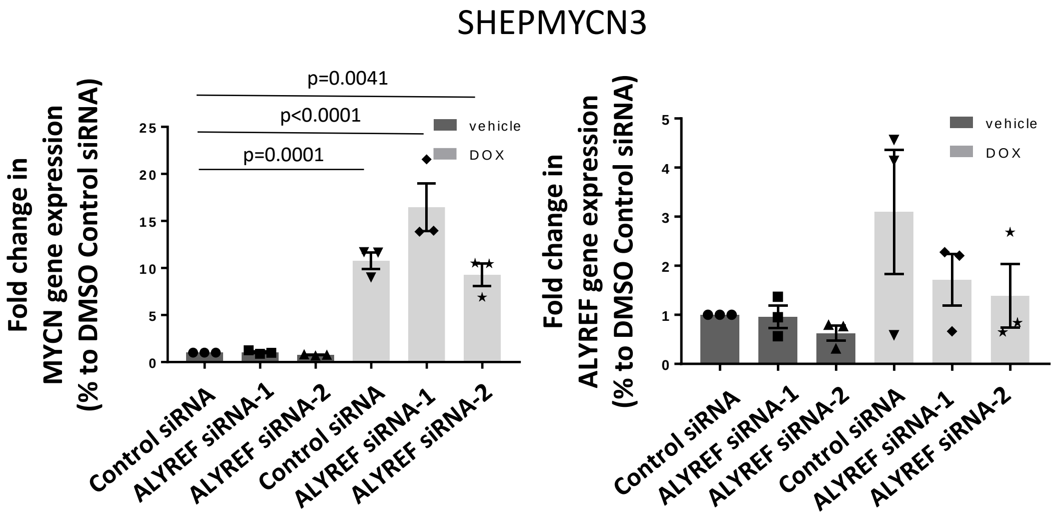

Supplementary Figure 5 (cont'd)

Supplementary Figure 5. ALYREF and MYCN form a transcriptional activator complex which upregulates USP3 expression, related to Fig. 5

(a) Genome-wide profile of ALYREF-chromatin interactions in SK-N-BE2C cells using chromatin immunoprecipitation sequencing (ChIP-seq). A total of 3598 ALYREF binding sites (FDR< 0.05) were identified, at intergenic (n=3375), intronic (n=210) regions and promoter proximal (n=13) positions. (b) ChIP assays (n=3 per group) were performed with a control IgG (IgG) or anti-ALYREF antibody, followed by PCR with primers targeting the negative control region (Control; 2000 bp downstream of TSS) or the *USP3* gene target sequence (USP3) containing the ALYREF binding sites. Fold enrichment was calculated as the difference in cycle thresholds obtained with the anti–ALYREF antibody and with the control IgG antibody. Differences in fold enrichment was compared to negative control (Control). Two-sided unpaired Student's t-tests were performed to derive p-values. (c) Schematic of 3C assay design at *USP3* gene showing locations of BamH1 and HindIII restriction sites and DNA fragments containing the MYCN binding site at the *USP3* promoter region (red, 0 bp) and that containing the ALYREF peak (green, + 6800bp from TSS). (d) Representative agarose gel from 3C assay showing PCR product of MYCN:ALYREF interaction in SK-N-BE(2)C cells (PCR Amplicon #1, lanes 1-8) and the positive control non-cross-linked DNA (PCR Amplicon #2, lanes 9-16). Genomic DNA, undigested genomic DNA from SK-N-BE(2)C cells; Genomic DNA +BamH1, BamH1 digested genomic DNA from SK-N-BE(2)C cells; Genomic DNA +BamH1 and HindIII, BamH1 and HindIII digested genomic DNA from SK-N-BE(2)C cells; Blank, no genomic DNA (water control); 3C product BamH1, product of PCR reaction following BamH1 digestion; 3C product BamH1 and Hind III, product of PCR reaction following BamH1 and HindIII digestion; 3C product BamH1 – ligase, ligase control for PCR reaction following BamH1 digestion; 3C product BamH1 and Hind III-ligase, ligase control for PCR reaction following BamH1 and HindIII digestion. The positions of molecular size standards (in base pairs) are shown to the left of the gel. (e) Electropherogram traces of a representative DNA Bioanalyzer run for 3C assay. FU=Fluorescence Unit; UM = Upper marker; LM = Lower marker peak. Arrows indicate the 3C PCR amplicon peaks. (f) Q-PCR analysis (n=3 per group) of *MYCN* and *ALYREF* expression following siRNA-mediated ALYREF knockdown for 72 hours with (DOX; 2µg/ml) and without doxycycline (DMSO as vehicle) for SHEPMYCN3 cells. Differences in expression levels were compared to the DMSO treated control siRNA (siControl). Two-sided one-way ANOVA was performed to derive p-values. P values are shown in comparison to the DMSO treated control siRNA (siControl). All other p values are listed in Source Data. Respective p values (p) on all figures are displayed. Comparisons were not significant unless otherwise noted. Data is representative of three independent experiments with similar results in d and e. Data are shown as mean ± s.e.m. (error bars) and representative of three independent experiments in b and f.

Supplementary Figure 6

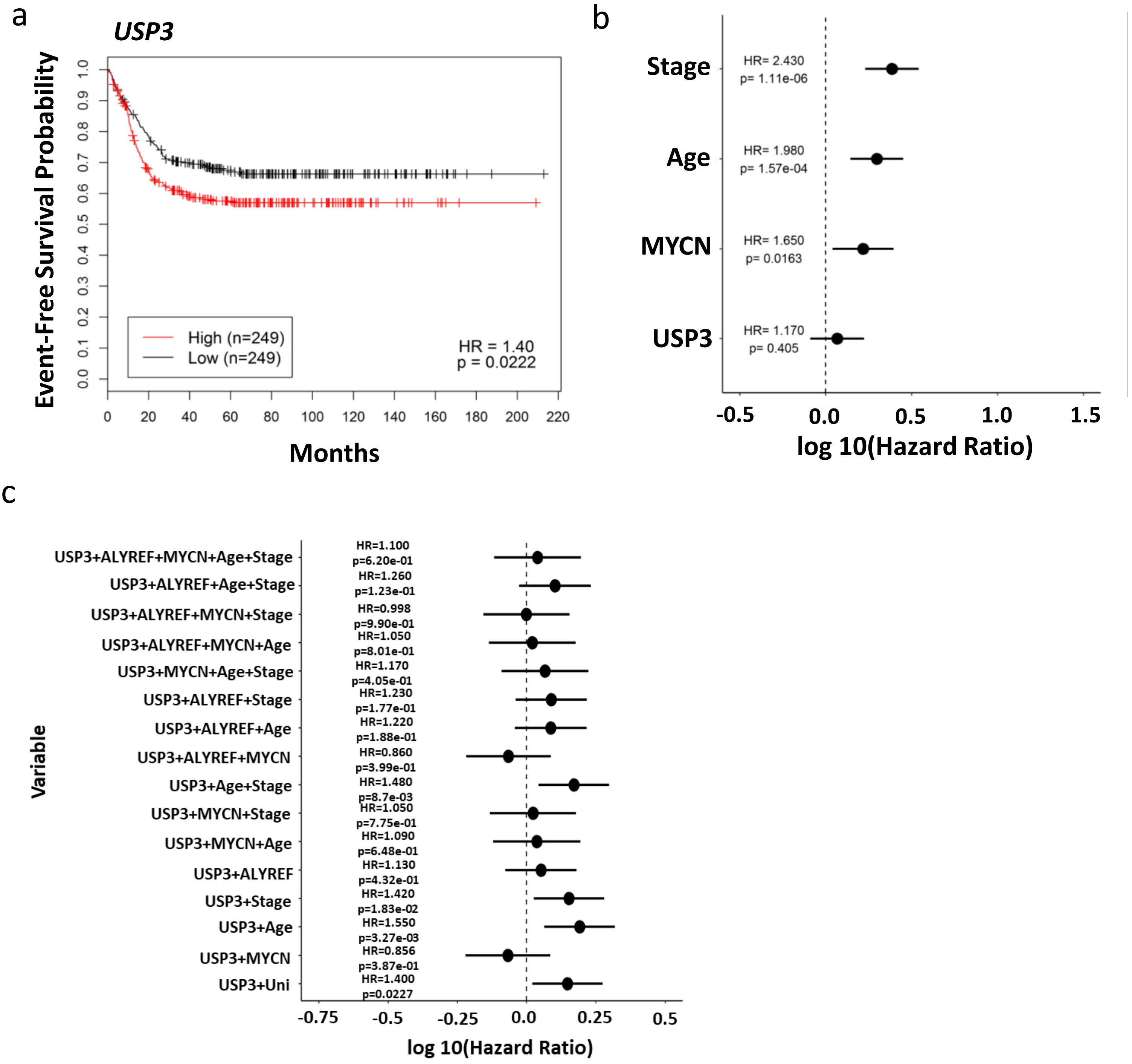

Supplementary Figure 6 (cont'd)

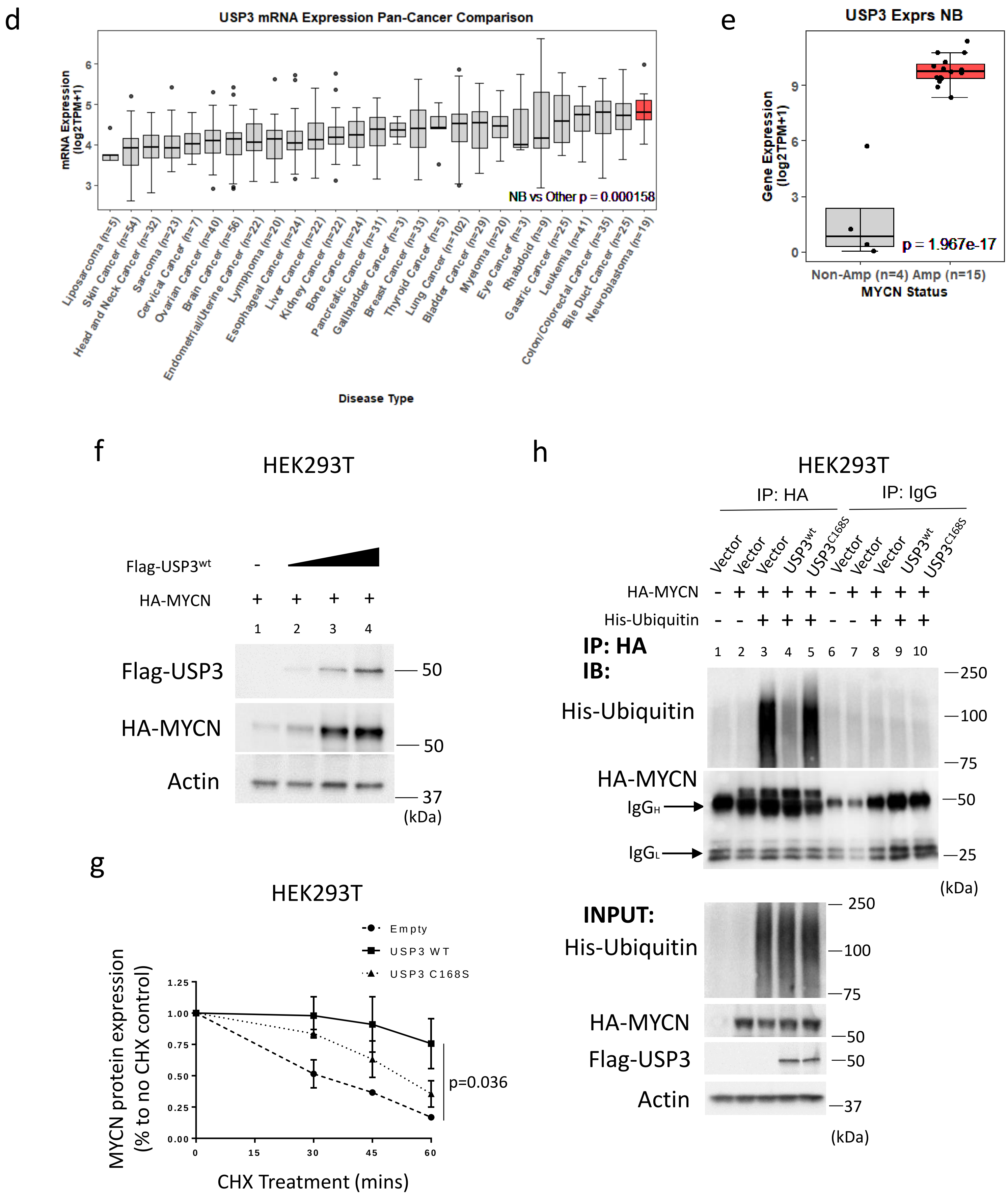

Supplementary Figure 6 (cont'd)

Supplementary Figure 6. USP3 expression associates with MYCN amplification and predicts poor patient survival, related to Fig. 6

(a) Kaplan-Meier survival curve showing the event-free survival probability of patients in the SEQC neuroblastoma cohort (n=498) when dichotomized by median *USP3* gene expression (RNA-Seq). Hazard Ratio's (HR) and two-sided log-rank p-values are presented from a univariate CoxPH model. (b) Multivariate event-free survival analysis dichotomized by stage of disease, age of patient at diagnosis, *MYCN* expression, and *USP3* expression using cox regression modelling. The p-values were obtained using two-sided log-rank tests. (c) Multivariate event-free survival analysis dichotomized by stage of disease, age of patient at diagnosis, *MYCN* expression, *ALYREF* expression and *USP3* expression using cox regression modelling. The p-values were obtained using two-sided log-rank tests. (d) *USP3* gene expression in transcripts per million (TPM) across 27 cancer types using the Cancer Cell Line Encyclopedia (CCLE) database, p-value is from a two-sample t-test between neuroblastoma cell lines and an aggregate of all other cell lines, samples are ranked by mean expression. (e) *USP3* gene expression between *MYCN*-non-amplified (n=4) and *MYCN*-amplified (n=15) cell lines using the CCLE database, p-value is from a two-sample t-test. Differences in *USP3* expression across the cell lines were compared using one-way ANOVA. (f) Immunoblot of HEK293T cells expressing HA-MYCN and either empty vector control ("-") or increasing amount of Flag-$USP3^{wt}$ at 24 hours. (g) Densitometry analysis (n=3 per group) of CHX assay. MYCN protein levels were normalized by actin, the ratio of MYCN protein/actin protein were artificially set as 1.0 for samples untreated with CHX to obtain half-life ($T_{1/2}$) of MYCN. p-value is from a two-sided unpaired t-test for 60 mins timepoint. (h) HEK293T cells expressing HA-MYCN, His-Ubiquitin, wild-type ($USP3^{wt}$) and mutant ($USP3^{C168S}$) USP3 proteins or empty vector (Vector) were treated with MG132 for 4 hours. Cells were then subjected to HA-MYCN immunoprecipitation and immunoblot analyses for ubiquitination. Respective p-values (p) on all figures are displayed. Comparisons were not significant unless otherwise noted. Data is representative of three independent experiments with similar results in f and h. Data are shown as mean ± s.e.m. (error bars) and representative of three independent experiments in g. The dot (measure of centre for the error bars) in each row represents the mean hazard ratio, while the error bars represent the 95% confidence intervals for the presented mean hazard ratio in b and c. For each boxplot, the line in the middle of the box represents the median expression value, and the upper/lower bounds of the boxes represent the interquartile range of all expression values (3rd quartile and 1st quartile respectively) in d and e. The whiskers represent 1.5x the interquartile range of all expression values, from the upper/lower bounds of the box in d and e.

Supplementary Figure 7

a

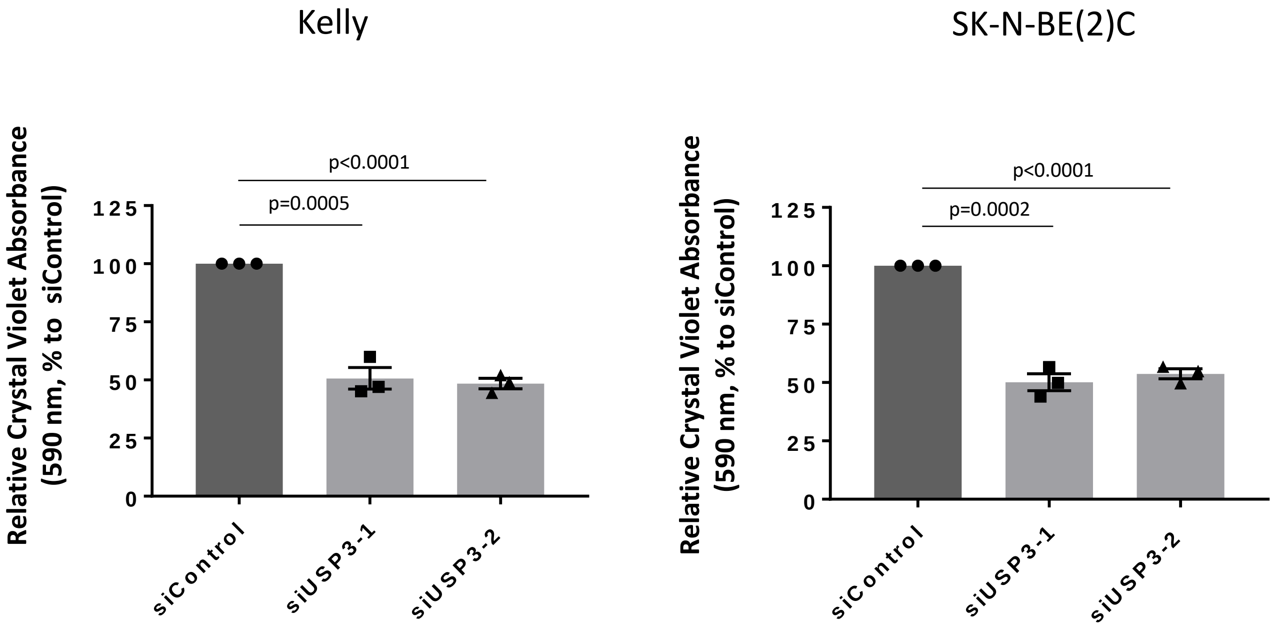

Supplementary Figure 7 (cont'd)

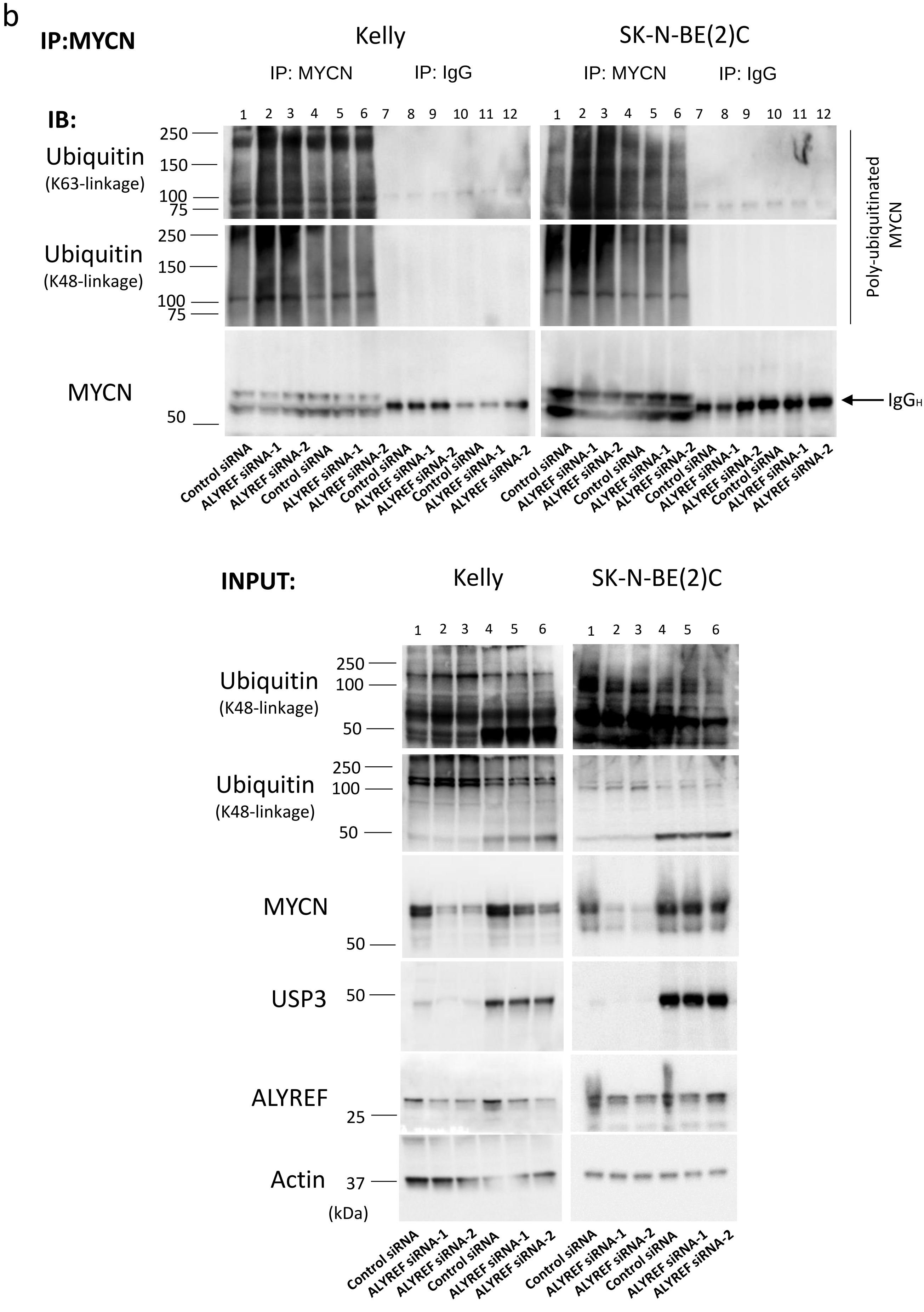

Supplementary Figure 7 (cont'd)

Supplementary Figure 7. USP3 is responsible for ALYREF-induced MYCN stability, related to Fig. 7
(a) Quantification of colony forming assay (n=3 per group) for Kelly and SK-N-BE(2)C cells transfected with USP3 siRNA-1, USP3 siRNA-2 or Control siRNA (siControl) based on crystal violet absorbance (590nm). Differences in colony formation were compared to the control siRNA (siControl) expressing cells. Two-sided unpaired Student's t-tests were performed to derive p-values. (b) Stable Kelly and SK-N-BE(2)C cells overexpressing USP3 (USP3) or Vector control (Vector) were co-transfected with ALYREF siRNA-1 or ALYREF siRNA-2 or control siRNA, followed by treatment with MG132 for 4 hours. Cells were then subjected to endogenous MYCN immunoprecipitation and immunoblot analyses for K-63 and K-48-linked ubiquitination. Respective p values (p) are displayed. Comparisons were not significant unless otherwise noted. Data are shown as mean ± s.e.m. (error bars) and representative of three independent experiments in a. Data is representative of two independent experiments with similar results in b.

Supplementary Figure 8

a

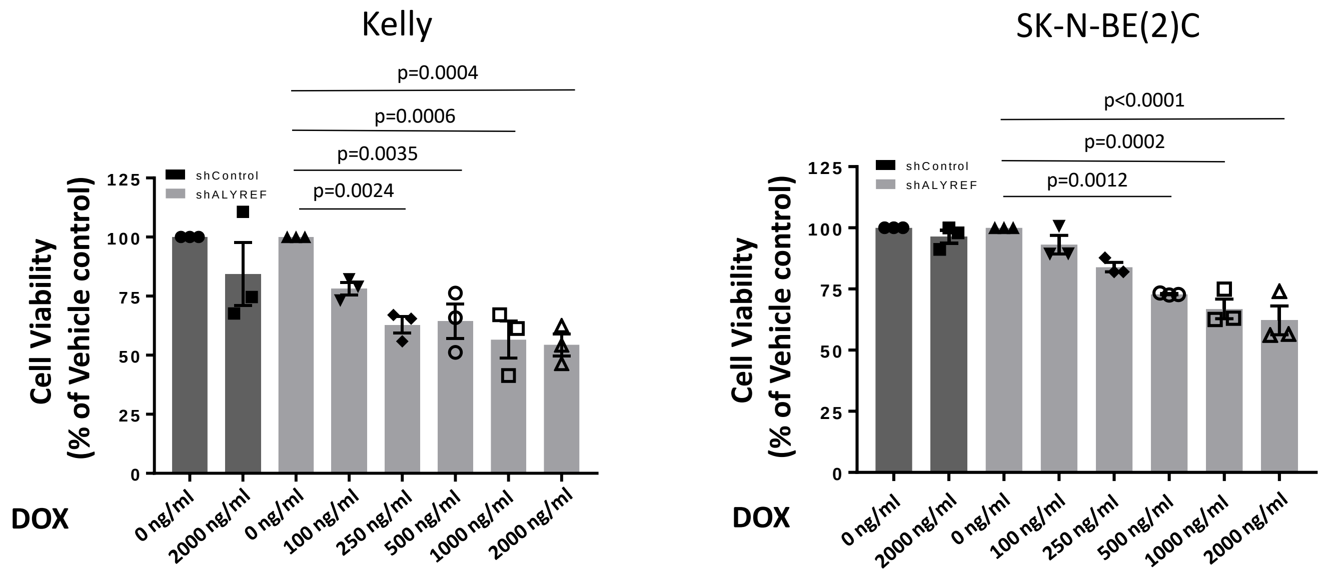

b

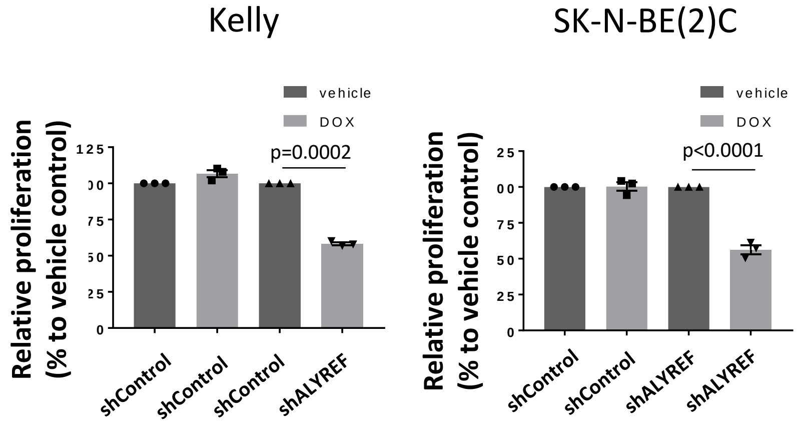

Supplementary Figure 8 (cont'd)

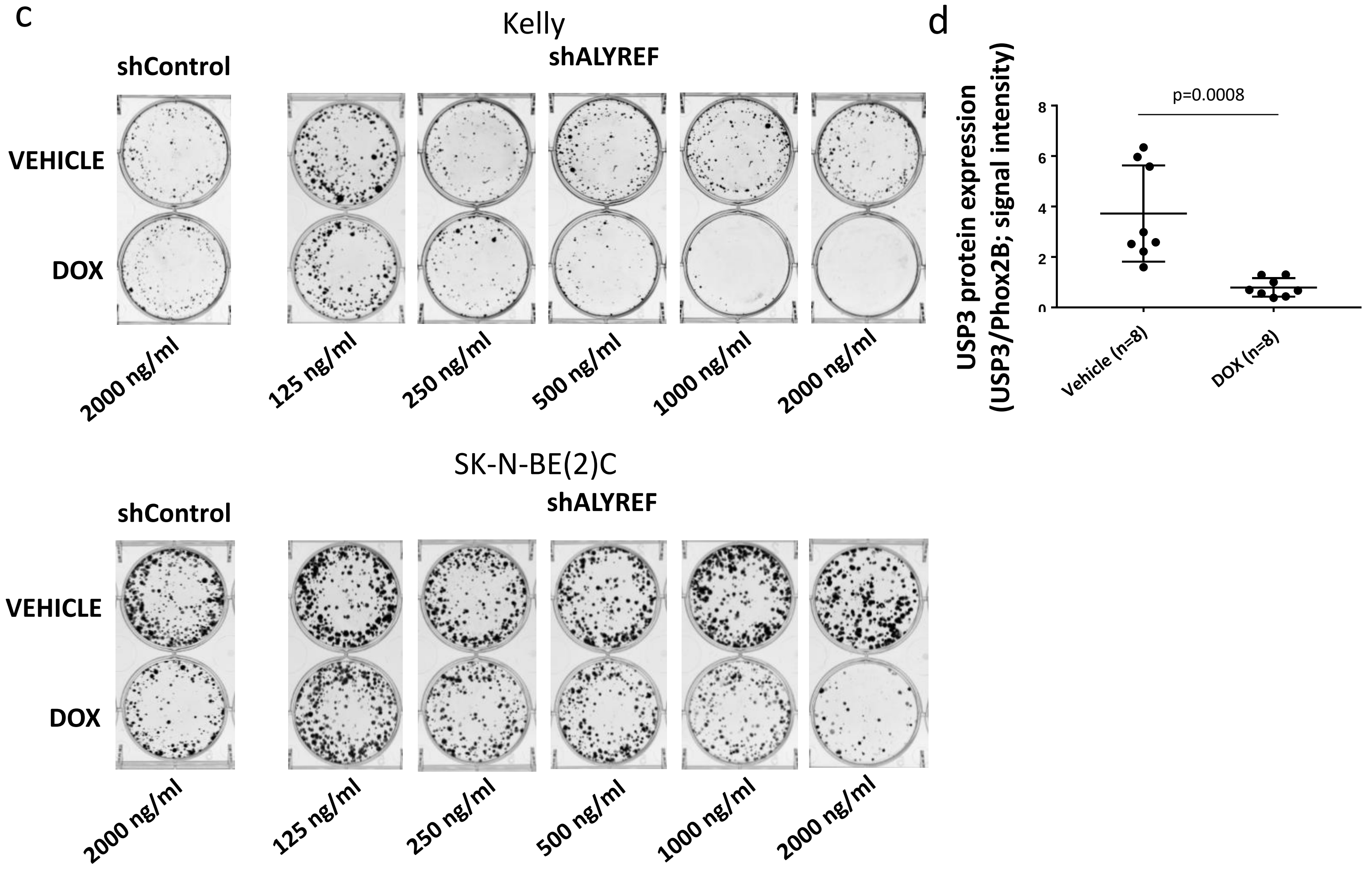

Supplementary Figure 8. ALYREF is required for the growth and tumorigenicity of MYCN-amplified neuroblastoma cells, related to Fig. 8

(a) Kelly and SK-N-BE(2)C cells expressing Control shRNA (shControl) or ALYREF shRNA (shALYREF) were treated with increasing doses of doxycycline (DOX) for 72 hours, then subjected to cell viability measurements (n=3 per group). Differences in cell viability were compared to the vehicle control shRNA (Vehicle). P values derived from two-sided one-way ANOVA test. (b) Kelly and SK-N-BE(2)C cells expressing ALYREF shRNA (shALYREF) were treated with doxycycline (DOX) for 72 hours, then subjected to cell proliferation measurements (n=3 per group). Differences in cell proliferation were compared to the vehicle control shRNA (Vehicle). Two-sided unpaired Student's t-tests were performed to derive p-values. (c) Kelly and SK-N-BE(2)C cells expressing Control shRNA (shControl) or ALYREF shRNA (shALYREF) were treated with increasing doses of doxycycline (DOX) for 12 days (Kelly) or 10 days (SK-N-BE(2)C), followed by colony formation assay. (d) Protein densitometry analysis of tumor samples (n=8 mice per treatment group) from SK-N-BE(2)C tumor xenografts, analyzed for USP3 expression. Phox2B was used as a neuroblastoma marker and loading control. Differences in USP3 protein expressions were compared to the vehicle treated (Vehicle) ALYREF shRNA subgroup. Two-sided unpaired Student's t-tests were performed to derive p-values. Respective p-values (p) on all figures are displayed. Comparisons were not significant unless otherwise noted. Data are shown as mean ± s.e.m. (error bars) and representative of three independent experiments in a and b. Data is show as mean ± s.e.m. (error bars) of mice (n=8) per treatment in d. Data is representative of three independent experiments with similar results in c.

gene_id	gene_short_name	chr	start	end	baseMean	log2FoldChange	lfcSE	stat	p-value	p.adj
ENSG00000261514	RP11-527L4.2.1	chr17	42015730	42016327	251.9986231	3.328336286	0.297924849	11.17173105	5.61E-29	7.87E-26
ENSG00000108821	COL1A1	chr17	48260649	48278993	261396.8104	2.127430162	0.296215653	7.182031542	6.87E-13	4.53E-11
ENSG00000238815	snoU13	chr17	49175534	49175639	0.72124544	2.09834227	0.809804215	2.591172323	0.009564958	0.029559774
ENSG00000180340	FZD2	chr17	42634826	42636907	298.8474298	1.811711702	0.31094428	5.826483455	5.66E-09	1.31E-07
ENSG00000244610	AC110921.1	chr17	65453139	65453406	1.117235071	1.809636578	0.559984422	3.231583783	0.001231062	0.005423188
ENSG00000207127	Y_RNA	chr17	47494105	47494212	0.514012089	1.721522739	0.479451069	3.590611956	0.000329902	0.001781513
ENSG00000200063	SNORA30	chr17	76396015	76396144	0.658235538	1.643773503	0.542932186	3.027585294	0.002465161	0.009697631
ENSG00000011028	MRC2	chr17	60704761	60770956	4957.03105	1.636450091	0.261979358	6.246484856	4.20E-10	1.31E-08
ENSG00000141756	FKBP10	chr17	39968961	39979465	11347.63342	1.632819026	0.201680149	8.096082025	5.68E-16	7.16E-14
ENSG00000166292	TMEM100	chr17	53796989	53809482	336.3662257	1.608022494	0.339749365	4.73296688	2.21E-06	2.44E-05
ENSG00000108849	PPY	chr17	42018171	42019833	10.30567293	1.59400915	0.539659835	2.953729456	0.003139591	0.011842312
ENSG00000214105	AC105337.1	chr17	77681074	77682784	1.213630057	1.429643973	0.500660927	2.855513375	0.004296731	0.015384662
ENSG00000221462	SNORA76	chr17	62223696	62223831	47.80233476	1.364290111	0.253795496	5.375548953	7.63E-08	1.27E-06
ENSG00000238419	U7	chr17	45336723	45336786	2.101375978	1.357146184	0.414255596	3.276108273	0.001052482	0.004736624
ENSG00000221555	AC068669.1	chr17	38306388	38306475	2.094706083	1.34275338	0.36392797	3.689613029	0.000224595	0.00129734
ENSG00000167281	RBFOX3	chr17	77085426	77613550	581.6353739	1.268714697	0.393846392	3.221343961	0.001275909	0.005587005
ENSG00000141337	ARSG	chr17	66255322	66418872	267.671267	1.265737288	0.221276632	5.720157955	1.06E-08	2.29E-07
ENSG00000200842	Y_RNA	chr17	60114193	60114306	2.655291049	1.226586147	0.408106646	3.005552982	0.002650984	0.010312933
ENSG00000064195	DLX3	chr17	48067368	48072588	8.721615062	1.187517047	0.451982821	2.627349961	0.008605278	0.027124962
ENSG00000241913	RP5-1073F15.1.1	chr17	57989557	57989727	2.206327477	1.091365713	0.461072339	2.367016236	0.017932146	0.049171484
ENSG00000242931	RP11-666A8.1.1	chr17	74426498	74427206	2.438773641	1.083771449	0.389515446	2.78235808	0.005396546	0.018534173
ENSG00000236194	AC003104.1.1	chr17	40423351	40424701	12.80891595	1.060655452	0.224381869	4.727010495	2.28E-06	2.50E-05
ENSG00000136457	CHAD	chr17	48541856	48546327	16.00415097	1.045038161	0.339030694	3.082429345	0.002053185	0.00831558
ENSG00000183010	PYCR1	chr17	79890268	79895161	10141.80155	1.04070762	0.149325788	6.9693764	3.18E-12	1.76E-10
ENSG00000249406	RP11-893F2.5.1	chr17	48286332	48291820	9.334340201	1.027966192	0.30028021	3.423356443	0.000618529	0.003021554
ENSG00000011052	NME2	chr17	49230919	49249108	46005.59464	1.024423125	0.15195377	6.741676257	1.57E-11	7.34E-10
ENSG00000239672	NME1	chr17	49230896	49239789	46005.59464	1.024423125	0.15195377	6.741676257	1.57E-11	7.34E-10
ENSG00000243678	NME1-NME2	chr17	49230950	49249105	46005.59464	1.024423125	0.15195377	6.741676257	1.57E-11	7.34E-10
ENSG00000242889	AC007431.1	chr17	55695468	55695741	2.611509601	1.011904022	0.355315771	2.847900671	0.004400866	0.015692676
ENSG00000163597	AC090699.1.1	chr17	74553851	74561430	5412.445252	0.982529206	0.156280368	6.286965027	3.24E-10	1.04E-08
ENSG00000200185	SNORD1C	chr17	74554872	74554950	5412.445252	0.982529206	0.156280368	6.286965027	3.24E-10	1.04E-08
ENSG00000213326	RPS7P11	chr17	44798947	44799533	2.485146076	0.966523327	0.246570261	3.919869835	8.86E-05	0.000587964
ENSG00000251085	RP11-893F2.6.1	chr17	48292054	48292781	15.90592059	0.919436476	0.310193836	2.96407075	0.003035985	0.011535034
ENSG00000200651	Y_RNA	chr17	75438190	75438295	5.502935319	0.903753762	0.310589792	2.90979866	0.003616617	0.013325973
ENSG00000108932	SLC16A6	chr17	66263166	66287405	109.7782645	0.872711946	0.230023984	3.794004139	0.000148237	0.000910062
ENSG00000232457	AC037487.1	chr17	62950241	62952716	9.726789137	0.855942521	0.234890232	3.644010709	0.000268422	0.001501542
ENSG00000182963	GJC1	chr17	42875815	42908179	3505.622117	0.851908461	0.167809568	5.076638178	3.84E-07	5.32E-06
ENSG00000141579	ZNF750	chr17	80787310	80797931	36.50567285	0.835559167	0.170291313	4.906645878	9.26E-07	1.14E-05
ENSG00000252042	Y_RNA	chr17	73141764	73141857	51.25391283	0.822616595	0.228716627	3.596662852	0.000322326	0.001747845
ENSG00000126368	NR1D1	chr17	38249039	38256978	1364.916782	0.805447367	0.281467927	2.861595544	0.004215144	0.015154655
ENSG00000141696	LEPREL4	chr17	39958198	39968855	3340.051552	0.80027984	0.11884486	6.733819525	1.65E-11	7.71E-10
ENSG00000235935	AC111170.3.1	chr17	75424896	75427072	14.56624559	0.797292409	0.271613328	2.935395011	0.003331233	0.012450557
ENSG00000171282	BAHCC1	chr17	79373539	79433357	4483.247407	0.763109474	0.231145439	3.301425623	0.000961948	0.004392608
ENSG00000229070	AC111170.2.1	chr17	75440785	75443249	96.98820901	0.756310487	0.177723504	4.255545661	2.09E-05	0.000170933
ENSG00000239246	RP11-464D20.2.1	chr17	60593681	60594128	3.062018291	0.753087224	0.239208507	3.148245988	0.001642534	0.006892784
ENSG00000136492	BRIP1	chr17	59759984	59940755	1428.893684	0.750363453	0.279746082	2.682301924	0.007311744	0.023680049
ENSG00000199753	SNORD104	chr17	62223442	62223512	175.5032451	0.741494377	0.197074558	3.762506872	0.000168219	0.001013286
ENSG00000201838	SNORD1A	chr17	74557715	74557787	11.70026999	0.701242401	0.211358473	3.317787025	0.000907336	0.004185155
ENSG00000131477	RAMP2	chr17	40913211	40915055	2386.983961	0.696806816	0.105709489	6.591714911	4.35E-11	1.81E-09
ENSG00000131471	AOC3	chr17	41003200	41010138	1701.524206	0.666774472	0.228206584	2.921802086	0.003480126	0.01290914
ENSG00000197291	AC100793.1	chr17	40905950	40913275	647.3539732	0.612288754	0.162389844	3.770486738	0.000162929	0.000985005
ENSG00000187013	C17orf82	chr17	59489111	59490641	46.4665501	0.596561396	0.191586419	3.113797927	0.001846959	0.007617402
ENSG00000214578	HMGN2P42	chr17	40904897	40905817	538.3220098	0.582466944	0.165662592	3.515983535	0.000438128	0.002263062
ENSG00000172809	RPL38	chr17	72199720	72206676	38050.4374	0.570969278	0.18351341	3.111321827	0.001862518	0.007664236
ENSG00000175711	B3GNTL1	chr17	80901671	81009686	883.7321431	0.568711656	0.109352982	5.200696362	1.99E-07	3.01E-06
ENSG00000131097	HIGD1B	chr17	42925278	42927840	136.2825446	0.568625817	0.240403507	2.365297512	0.018015592	0.049363182
ENSG00000123700	KCNJ2	chr17	68164813	68176160	217.1226226	0.561151561	0.225679902	2.486493283	0.0129009	0.037842434
ENSG00000183979	NPB	chr17	79860071	79860780	194.0958438	0.550665888	0.223406453	2.464861153	0.013706635	0.039748698
ENSG00000184009	ACTG1	chr17	79476998	79479827	173506.1296	0.539884564	0.123158926	4.383641364	1.17E-05	0.000103986
ENSG00000121057	AKAP1	chr17	55162550	55198710	7624.151018	0.533661495	0.125366912	4.256796996	2.07E-05	0.000170248
ENSG00000250506	CDK3	chr17	73996986	74002078	4568.599571	0.531107694	0.105197347	5.048679547	4.45E-07	6.02E-06
ENSG00000257949	TEN1	chr17	73975300	73996667	4568.599571	0.531107694	0.105197347	5.048679547	4.45E-07	6.02E-06
ENSG00000261408	RP11-685I11.1.1	chr17	73975311	74002080	4568.599571	0.531107694	0.105197347	5.048679547	4.45E-07	6.02E-06
ENSG00000169710	FASN	chr17	80036214	80056106	38563.29846	0.526080433	0.120279082	4.373831457	1.22E-05	0.000107981
ENSG00000183077	AFMID	chr17	76183397	76203782	3985.335488	0.502696625	0.107087915	4.69424233	2.68E-06	2.88E-05
ENSG00000108984	MAP2K6	chr17	67410837	67538451	742.5375126	0.490640854	0.146070255	3.358937486	0.000782428	0.003692131
ENSG00000224877	C17orf89	chr17	79213110	79215098	1246.838492	0.487898041	0.145812822	3.346057195	0.000819695	0.003838681
ENSG00000169738	DCXR	chr17	79993756	79995573	6205.416367	0.479041043	0.138018002	3.470859133	0.000518796	0.002606273
ENSG00000183684	ALYREF	chr17	79845712	79849462	9659.134541	0.478751068	0.149491821	3.202523474	0.001362292	0.005897282
ENSG00000108379	WNT3	chr17	44841693	44896082	267.4086356	0.476866506	0.184001155	2.591649521	0.009551702	0.029528818
ENSG00000108924	HLF	chr17	53342320	53402426	1293.652958	0.470764107	0.142789396	3.296912243	0.00097754	0.004452656
ENSG00000141569	TRIM65	chr17	73885040	73893084	2486.760141	0.446748803	0.122055162	3.660220488	0.000251998	0.00142519

Supplementary Table 1. List of genes differently expressed on the 17q21-ter locus in patients with MYCN -amplification vs no amplification, related to Fig. 1

DESeq2 (differential gene expression) output for those genes that were significantly more highly expressed in patients with MYCN-amplification on the 17q21-ter locus in the TARGET neuroblastoma patient cohort. Two-sided Wald Chi-Squared tests between patient groups were first performed to generate p-values, followed by use of the Benjamini-Hochberg method to adjust p-values for multiple comparisons (DeSeq2 methodology).

gene_id	gene_name1	gene_name2	method	n	df	t-statistic	r	95.conf.int.low	95.conf.int.high	p-value	p.adj
ENSG00000183010	PYCR1	MYCN	pearson	154	152	11.08525909	0.6686	0.570809626	0.747701595	2.67E-21	2.63E-18
ENSG00000261514	RP11-527L4.2.1	MYCN	pearson	154	152	8.250473537	0.5562	0.436382489	0.656565584	6.99E-14	6.87E-11
ENSG00000250506	CDK3	MYCN	pearson	154	152	6.832740614	0.4847	0.353698717	0.597123227	1.89E-10	1.85E-07
ENSG00000257949	TEN1	MYCN	pearson	154	152	6.832740614	0.4847	0.353698717	0.597123227	1.89E-10	1.85E-07
ENSG00000261408	RP11-685I11.1.1	MYCN	pearson	154	152	6.832740614	0.4847	0.353698717	0.597123227	1.89E-10	1.85E-07
ENSG00000136492	BRIP1	MYCN	pearson	154	152	6.650486369	0.4748	0.342299413	0.588712925	4.95E-10	4.87E-07
ENSG00000182963	GJC1	MYCN	pearson	154	152	6.568897905	0.4702	0.337140157	0.584888856	7.60E-10	7.47E-07
ENSG00000089685	BIRC5	MYCN	pearson	154	152	6.563771926	0.4699	0.336814859	0.584647373	7.80E-10	7.67E-07
ENSG00000197291	AC100793.1	MYCN	pearson	154	152	6.378153335	0.4595	0.324943625	0.575804711	2.04E-09	2.01E-06
ENSG00000214578	HMGN2P42	MYCN	pearson	154	152	6.263909943	0.453	0.31754888	0.570266676	3.67E-09	3.61E-06
ENSG00000173894	CBX2	MYCN	pearson	154	152	6.246466062	0.452	0.316413885	0.569414623	4.01E-09	3.94E-06
ENSG00000163597	AC090699.1.1	MYCN	pearson	154	152	6.009048683	0.4381	0.300812338	0.557647017	1.33E-08	1.30E-05
ENSG00000200185	SNORD1C	MYCN	pearson	154	152	6.009048683	0.4381	0.300812338	0.557647017	1.33E-08	1.30E-05
ENSG00000186185	KIF18B	MYCN	pearson	154	152	5.971843048	0.4359	0.298341614	0.555773935	1.60E-08	1.57E-05
ENSG00000089558	KCNH4	MYCN	pearson	154	152	5.915887499	0.4326	0.294612701	0.552942048	2.11E-08	2.07E-05
ENSG00000011052	NME2	MYCN	pearson	154	152	5.793766681	0.4253	0.286420336	0.546699404	3.83E-08	3.77E-05
ENSG00000239672	NME1	MYCN	pearson	154	152	5.793766681	0.4253	0.286420336	0.546699404	3.83E-08	3.77E-05
ENSG00000243678	NME1-NME2	MYCN	pearson	154	152	5.793766681	0.4253	0.286420336	0.546699404	3.83E-08	3.77E-05
ENSG00000125319	C17orf53	MYCN	pearson	154	152	5.768649353	0.4238	0.284726206	0.545404844	4.33E-08	4.26E-05
ENSG00000141696	LEPREL4	MYCN	pearson	154	152	5.760106055	0.4233	0.284149263	0.544963693	4.52E-08	4.44E-05
ENSG00000131477	RAMP2	MYCN	pearson	154	152	5.622957575	0.415	0.274838428	0.537824259	8.76E-08	8.61E-05
ENSG00000094804	CDC6	MYCN	pearson	154	152	5.561475519	0.4112	0.27063476	0.534588531	1.17E-07	0.00011544
ENSG00000108984	MAP2K6	MYCN	pearson	154	152	5.525775488	0.409	0.268185489	0.532699656	1.39E-07	0.00013678
ENSG00000167900	TK1	MYCN	pearson	154	152	5.512885436	0.4082	0.267299635	0.532015838	1.48E-07	0.000145395
ENSG00000136450	SRSF1	MYCN	pearson	154	152	5.508872713	0.408	0.267023703	0.531802766	1.51E-07	0.000148184
ENSG00000131747	TOP2A	MYCN	pearson	154	152	5.502437175	0.4076	0.266581006	0.53146085	1.55E-07	0.000152765
ENSG00000154920	EME1	MYCN	pearson	154	152	5.497875928	0.4073	0.26626712	0.53121837	1.59E-07	0.000156096
ENSG00000185504	C17orf70	MYCN	pearson	154	152	5.355921573	0.3984	0.256448843	0.523611651	3.09E-07	0.000303743
ENSG00000184009	ACTG1	MYCN	pearson	154	152	5.275207577	0.3934	0.25082381	0.519234375	4.49E-07	0.000441359
ENSG00000121057	AKAP1	MYCN	pearson	154	152	5.26808231	0.3929	0.250325779	0.518846138	4.64E-07	0.000456083
ENSG00000183077	AFMID	MYCN	pearson	154	152	5.262231398	0.3926	0.249916645	0.518527117	4.77E-07	0.000468531
ENSG00000141556	TBCD	MYCN	pearson	154	152	5.108848646	0.3828	0.239134619	0.510092841	9.59E-07	0.000942633
ENSG00000198933	TBKBP1	MYCN	pearson	154	152	5.028378178	0.3777	0.233434918	0.505613077	1.38E-06	0.001353056
ENSG00000012048	BRCA1	MYCN	pearson	154	152	4.892684309	0.3689	0.223757893	0.497973515	2.51E-06	0.002467911
ENSG00000130935	NOL11	MYCN	pearson	154	152	4.817044307	0.3639	0.218328273	0.493668361	3.49E-06	0.003433867
ENSG00000011258	MBTD1	MYCN	pearson	154	152	4.808313207	0.3633	0.217699925	0.49316927	3.63E-06	0.003566539
ENSG00000183684	ALYREF	MYCN	pearson	154	152	4.797150097	0.3626	0.21689607	0.492530511	3.81E-06	0.003743409
ENSG00000229070	AC111170.2.1	MYCN	pearson	154	152	4.788224476	0.362	0.216252949	0.49201926	3.96E-06	0.003890908
ENSG00000011143	MKS1	MYCN	pearson	154	152	4.597747818	0.3494	0.202447121	0.480998227	8.93E-06	0.0087742
ENSG00000068489	PRR11	MYCN	pearson	154	152	4.565400437	0.3473	0.200087358	0.479105604	1.02E-05	0.010051189
ENSG00000169683	LRRC45	MYCN	pearson	154	152	4.510306228	0.3436	0.196058215	0.475868064	1.29E-05	0.012649366
ENSG00000252042	Y_RNA	MYCN	pearson	154	152	4.490472143	0.3422	0.194604653	0.474698219	1.40E-05	0.013734486
ENSG00000189159	HN1	MYCN	pearson	154	152	4.361050788	0.3335	0.185080703	0.467008657	2.38E-05	0.023354898
ENSG00000175711	B3GNTL1	MYCN	pearson	154	152	4.349218202	0.3327	0.184206608	0.466300777	2.49E-05	0.02450332
ENSG00000161547	SRSF2	MYCN	pearson	154	152	4.347237614	0.3325	0.184060245	0.46618221	2.51E-05	0.024700773
ENSG00000207556	MIR636	MYCN	pearson	154	152	4.347237614	0.3325	0.184060245	0.46618221	2.51E-05	0.024700773
ENSG00000108825	AARSD1	MYCN	pearson	154	152	4.292594974	0.3288	0.180016094	0.462902091	3.13E-05	0.030792115
ENSG00000183048	MRPL12	MYCN	pearson	154	152	4.278720747	0.3279	0.178987385	0.462066491	3.31E-05	0.032554535
ENSG00000184247	AC100791.1	MYCN	pearson	154	152	4.260011632	0.3266	0.177599005	0.460937939	3.57E-05	0.035084908
ENSG00000221462	SNORA76	MYCN	pearson	154	152	4.258501233	0.3265	0.177486861	0.460846742	3.59E-05	0.035297217
ENSG00000169710	FASN	MYCN	pearson	154	152	4.224716739	0.3242	0.17497612	0.45880341	4.11E-05	0.040380985
ENSG00000171634	BPTF	MYCN	pearson	154	152	4.191891306	0.3219	0.172532448	0.456811773	4.68E-05	0.04598768
ENSG00000182481	KPNA2	MYCN	pearson	154	152	4.191891306	0.3219	0.172532448	0.456811773	4.68E-05	0.04598768
ENSG00000222881	Y_RNA	MYCN	pearson	154	152	4.176314369	0.3208	0.17137139	0.45586449	4.97E-05	0.04890213

Supplementary Table 2. Correlation analysis of genes located on 17q21-ter locus with MYCN -amplification, related to Fig.1

Results of Pearson correlations for those genes on the 17q21-ter locus that were both significantly and positively correlated with MYCN gene expression. Parametric Pearson correlation tests were conducted between each gene's expression and its respective ploidy to derive correlation coefficients (r). T-statistics were then calculated (where degrees of freedom = 88) from which p-values were generated from, via null hypothesis testing (two-sided). Confidence intervals, effect sizes (n), degrees of freedom and p-values are provided. Finally, p-values were adjusted for multiple comparisons using the Benjamini-Hochberg method.

gene_id	gene_short_name	locus	Beta.Coeff.	Hazard Ratio	95.conf.int.low	95.conf.int.high	LogRank.P.Val	LogRank Statistic	Wald.P.Val	Wald.Z.Val	Wald Statistic
ENSG00000183010	PYCR1	chr17:79890268-79895161	1	2.8	1.7	4.5	1.10E-05	19	2.30E-05	4.2	18
ENSG00000256525	POLG2	chr17:62473903-62493184	0.82	2.3	1.4	3.7	0.00044	12	0.00063	3.4	12
ENSG00000260369	CTD-2526A2.2.1	chr17:78427534-78428529	0.82	2.3	1.4	3.6	0.00052	12	0.00074	3.4	11
ENSG00000238331	AC100791.2	chr17:77784477-77784573	0.81	2.3	1.3	3.8	0.0046	8	0.0024	3	9.2
ENSG00000203315	AC015815.2	chr17:75087900-75088024	0.78	2.2	1.4	3.5	0.00074	11	0.00091	3.3	11
ENSG00000241157	RP11-3K24.1.1	chr17:58322648-58323008	0.77	2.2	1.3	3.5	0.0011	11	0.0015	3.2	10
ENSG00000167085	PHB	chr17:47481413-47492246	0.75	2.1	1.3	3.4	0.0013	10	0.0016	3.2	9.9
ENSG00000182459	TEX19	chr17:80317122-80321652	0.73	2.1	1.3	3.3	0.0016	10	0.0019	3.1	9.7
ENSG00000186395	KRT10	chr17:38974368-38978847	0.71	2	1.3	3.3	0.0022	9.4	0.0027	3	9
ENSG00000141522	ARHGDIA	chr17:79825597-79829282	0.71	2	1.3	3.2	0.0024	9.2	0.003	3	8.8
ENSG00000213135	AC015815.6	chr17:75085985-75087063	0.69	2	1.3	3.2	0.0026	9.1	0.003	3	8.8
ENSG00000185298	CCDC137	chr17:79633760-79640937	0.67	2	1.2	3.1	0.004	8.3	0.0049	2.8	7.9
ENSG00000244610	AC110921.1	chr17:65453139-65453406	0.67	1.9	1.2	3.1	0.0044	8.1	0.0039	2.9	8.3
ENSG00000221462	SNORA76	chr17:62223696-62223831	0.66	1.9	1.2	3.1	0.004	8.3	0.0046	2.8	8
ENSG00000012048	BRCA1	chr17:41196311-41322290	0.67	1.9	1.2	3.1	0.0042	8.2	0.0049	2.8	7.9
ENSG00000167900	TK1	chr17:76170159-76183314	0.66	1.9	1.2	3.1	0.0045	8.1	0.0052	2.8	7.8
ENSG00000141560	FN3KRP	chr17:80674581-80685892	0.66	1.9	1.2	3.1	0.0049	7.9	0.006	2.7	7.5
ENSG00000181038	METTL23	chr17:74722939-74729961	0.63	1.9	1.2	3	0.0067	7.4	0.0076	2.7	7.1
ENSG00000094804	CDC6	chr17:38443884-38459171	0.63	1.9	1.2	3	0.0066	7.4	0.0077	2.7	7.1
ENSG00000108786	HSD17B1	chr17:40703983-40707857	0.62	1.9	1.2	2.9	0.0074	7.2	0.0083	2.6	7
ENSG00000068120	COASY	chr17:40714091-40718295	0.62	1.9	1.2	2.9	0.0075	7.1	0.0085	2.6	6.9
ENSG00000214401	AC217773.1	chr17:44270938-44274037	0.61	1.8	1.2	2.9	0.0081	7	0.0091	2.6	6.8
ENSG00000229070	AC111170.2.1	chr17:75440785-75443249	0.61	1.8	1.2	2.9	0.0085	6.9	0.0093	2.6	6.8
ENSG00000197723	HSPB9	chr17:40274755-40275371	0.61	1.8	1.2	2.9	0.0085	6.9	0.0095	2.6	6.7
ENSG00000131462	TUBG1	chr17:40761357-40767254	0.58	1.8	1.1	2.8	0.011	6.4	0.012	2.5	6.2
ENSG00000141378	PTRH2	chr17:57774666-57784987	0.59	1.8	1.1	2.8	0.011	6.5	0.012	2.5	6.3
ENSG00000141570	CBX8	chr17:77765930-77775482	0.59	1.8	1.1	2.9	0.011	6.5	0.012	2.5	6.4
ENSG00000141552	ANAPC11	chr17:79849598-79858363	0.58	1.8	1.1	2.8	0.011	6.4	0.013	2.5	6.2
ENSG00000182963	GJC1	chr17:42875815-42908179	0.57	1.8	1.1	2.8	0.013	6.1	0.015	2.4	6
ENSG00000198231	DDX42	chr17:61851566-61896676	0.58	1.8	1.1	2.8	0.013	6.2	0.015	2.4	6
ENSG00000154920	EME1	chr17:48450580-48458820	0.56	1.8	1.1	2.8	0.015	5.9	0.016	2.4	5.8
ENSG00000167862	ICT1	chr17:73008779-73017355	0.56	1.8	1.1	2.8	0.015	5.9	0.016	2.4	5.8
ENSG00000141543	EIF4A3	chr17:78109012-78120982	0.56	1.8	1.1	2.8	0.015	5.9	0.017	2.4	5.7
ENSG00000169689	STRA13	chr17:79976578-79980794	0.55	1.7	1.1	2.8	0.016	5.8	0.018	2.4	5.6
ENSG00000169718	DUS1L	chr17:80015747-80023680	0.55	1.7	1.1	2.7	0.017	5.7	0.019	2.4	5.5
ENSG00000011143	MKS1	chr17:56282798-56296966	0.55	1.7	1.1	2.7	0.018	5.6	0.019	2.3	5.5
ENSG00000141568	FOXK2	chr17:80477588-80562483	0.54	1.7	1.1	2.7	0.019	5.5	0.02	2.3	5.4
ENSG00000166292	TMEM100	chr17:53796989-53809482	0.53	1.7	1.1	2.7	0.02	5.4	0.022	2.3	5.3
ENSG00000200651	Y_RNA	chr17:75438190-75438295	0.54	1.7	1.1	2.7	0.02	5.4	0.022	2.3	5.2
ENSG00000228782	AC040934.1	chr17:45527592-45569849	0.53	1.7	1.1	2.7	0.021	5.3	0.022	2.3	5.2
ENSG00000087995	METTL2A	chr17:60501245-60527454	0.53	1.7	1.1	2.7	0.022	5.3	0.023	2.3	5.1
ENSG00000108423	TUBD1	chr17:57936850-57970296	0.53	1.7	1.1	2.7	0.022	5.2	0.023	2.3	5.1
ENSG00000167925	GHDC	chr17:40341105-40346550	0.53	1.7	1.1	2.7	0.022	5.2	0.023	2.3	5.2
ENSG00000207021	Y_RNA	chr17:79540515-79540628	0.53	1.7	1.1	2.7	0.022	5.2	0.023	2.3	5.1
ENSG00000240656	AC068594.1	chr17:75158031-75158389	0.54	1.7	1.1	2.7	0.026	4.9	0.023	2.3	5.2
ENSG00000179673	RPRML	chr17:45055522-45056614	0.53	1.7	1.1	2.7	0.022	5.2	0.024	2.3	5.1
ENSG00000182040	USH1G	chr17:72912175-72919351	0.53	1.7	1.1	2.7	0.022	5.3	0.024	2.3	5.1
ENSG00000204283	AC015804.1	chr17:75875108-75878659	0.53	1.7	1.1	2.7	0.022	5.2	0.024	2.3	5.1
ENSG00000129654	FOXJ1	chr17:74132424-74137380	0.52	1.7	1.1	2.7	0.024	5.1	0.026	2.2	4.9
ENSG00000133195	SLC39A11	chr17:70642085-71088853	0.52	1.7	1.1	2.7	0.024	5.1	0.026	2.2	5
ENSG00000183684	ALYREF	chr17:79845712-79849462	0.51	1.7	1.1	2.6	0.028	4.8	0.03	2.2	4.7
ENSG00000186185	KIF18B	chr17:43003359-43025082	0.51	1.7	1	2.6	0.028	4.8	0.03	2.2	4.7
ENSG00000228733	AC015815.8	chr17:75108842-75108930	0.5	1.7	1.1	2.6	0.029	4.8	0.03	2.2	4.7
ENSG00000171595	DNAI2	chr17:72270385-72311022	0.5	1.7	1	2.6	0.029	4.7	0.031	2.2	4.7
ENSG00000261514	RP11-527L4.2.1	chr17:42015730-42016327	0.49	1.6	1	2.6	0.031	4.6	0.032	2.1	4.6
ENSG00000108829	LRRC59	chr17:48458598-48474914	0.49	1.6	1	2.6	0.033	4.5	0.035	2.1	4.4
ENSG00000131469	RPL27	chr17:41150445-41154956	0.48	1.6	1	2.6	0.035	4.5	0.036	2.1	4.4
ENSG00000229944	AC004797.1.1	chr17:47500969-47502298	0.48	1.6	1	2.5	0.037	4.4	0.038	2.1	4.3
ENSG00000163597	AC090699.1.1	chr17:74553851-74561430	0.47	1.6	1	2.5	0.039	4.3	0.041	2	4.2
ENSG00000200185	SNORD1C	chr17:74554872-74554950	0.47	1.6	1	2.5	0.039	4.3	0.041	2	4.2
ENSG00000260248	RP11-143K11.1.1	chr17:71171621-71172772	0.48	1.6	1	2.6	0.039	4.3	0.042	2	4.2
ENSG00000169710	FASN	chr17:80036214-80056106	0.47	1.6	1	2.5	0.04	4.2	0.042	2	4.1
ENSG00000189159	HN1	chr17:73131342-73150778	0.47	1.6	1	2.5	0.04	4.2	0.042	2	4.1
ENSG00000200842	Y_RNA	chr17:60114193-60114306	0.47	1.6	1	2.5	0.041	4.2	0.042	2	4.1
ENSG00000198496	NBR2	chr17:41277608-41305688	0.47	1.6	1	2.5	0.04	4.2	0.043	2	4.1
ENSG00000169696	ASPSCR1	chr17:79935425-79975280	0.47	1.6	1	2.5	0.041	4.2	0.044	2	4.1
ENSG00000011052	NME2	chr17:49230919-49249108	0.47	1.6	1	2.5	0.043	4.1	0.044	2	4
ENSG00000239672	NME1	chr17:49230896-49239789	0.47	1.6	1	2.5	0.043	4.1	0.044	2	4
ENSG00000243678	NME1-NME2	chr17:49230950-49249105	0.47	1.6	1	2.5	0.043	4.1	0.044	2	4
ENSG00000224877	C17orf89	chr17:79213110-79215098	0.47	1.6	1	2.5	0.043	4.1	0.045	2	4
ENSG00000108588	CCDC47	chr17:61822610-61851088	0.46	1.6	1	2.5	0.044	4.1	0.046	2	4
ENSG00000182173	TSEN54	chr17:73511787-73520820	0.46	1.6	1	2.5	0.044	4	0.047	2	4
ENSG00000108384	RAD51C	chr17:56769933-56811703	0.46	1.6	1	2.5	0.045	4	0.047	2	4
ENSG00000237627	THA1P	chr17:76244573-76250497	0.46	1.6	1	2.5	0.045	4	0.047	2	4
ENSG00000131467	PSME3	chr17:40976442-40995775	0.46	1.6	1	2.5	0.046	4	0.048	2	3.9
ENSG00000159210	SNF8	chr17:47006677-47022479	0.46	1.6	1	2.5	0.046	4	0.048	2	3.9
ENSG00000136492	BRIP1	chr17:59759984-59940755	0.46	1.6	1	2.5	0.047	3.9	0.049	2	3.9

Supplementary Table 3. CoxPH analysis for genes located on 17q21-ter locus, related to Fig. 1

Results of Cox proportional hazard models for those genes on 17q21-ter locus whose expression significantly associated with poor outcome. Two-sided (for both log-rank and Wald (chi-squared) statistical tests).

Chromosome	Start	End	Gene	FE	#-log10FDR	Region	Distance from TSS
chr4	173483222	173485157	SCRG1	13.24372	34.38903	intron (NM_001329597, intron 1 of 3)	35049
chr11	71085591	71086541	SHANK2	8.03239	14.14267	intron (NM_012309, intron 7 of 24)	138730
chr11	24918985	24919309	LUZP2	7.39752	11.64372	intron (NM_001252008, intron 7 of 11)	422177
chr9	137102163	137102932	DPP7	7.11084	12.46696	intron (NR_045720, intron 8 of 12)	12196
chr16	3529428	3529845	CLUAP1	6.16223	13.47369	intron (NM_001330454, intron 9 of 12)	19742
chr15	63511230	63511496	USP3	6.15987	8.68653	intron (NM_001256702, intron 1 of 13)	6852
chr12	96825426	96826951	NEDD1	5.54025	19.49911	intron (NM_001306084, intron 65 of 67)	-81035
chr11	14160960	14161312	RRAS2	5.38001	5.96926	intron (NM_006108, intron 6 of 15)	197349
chr4	72529862	72530791	ADAMTS3	5.38001	5.96926	intron (NM_014243, intron 3 of 21)	38473
chr2	43514785	43515349	THADA	5.07807	5.48122	intron (NR_144316, intron 22 of 36)	80907
chr6	162010580	162011378	LOC105378098	5.04376	5.11835	intron (NM_013988, intron 2 of 8)	540002
chr1	227607675	227607915	ZNF678	4.79985	8.10689	intron (NR_102302, intron 2 of 5)	44276
chr4	183851488	183851869	STOX2	4.70751	4.30614	intron (NR_132761, intron 1 of 2)	53656
chr1	202172466	202172857	PTPRVP	4.70751	4.30614	intron (NR_002930, intron 6 of 21)	4610
chr1	246024758	246025035	SMYD3	4.50187	4.52818	intron (NM_001167740, intron 5 of 11)	392516
chr10	1238579	1239352	LINC00200	4.49814	3.98149	intron (NM_018702, intron 5 of 9)	79197
chr15	75530551	75530873	PTPN9	4.47649	7.6514	intron (NM_002833, intron 1 of 12)	48579
chr22	28083229	28083529	MIR3199-1	4.47056	3.93765	intron (NM_001145418, intron 12 of 22)	-162767
chr7	14211450	14211988	ETV1	4.41118	4.11032	intron (NM_145695, intron 22 of 23)	-220294
chr8	40645341	40645572	ZMAT4	4.37126	3.52779	intron (NM_001135731, intron 4 of 5)	252368
chr7	99597526	99597781	TMEM225B	4.37126	3.52779	promoter-TSS (NM_001195541)	-413
chr6	158169408	158169659	GTF2H5	4.37126	3.52779	intron (NM_207118, intron 1 of 2)	1186
chr15	56674674	56674941	ZNF280D	4.26651	3.87143	intron (NM_017661, intron 13 of 21)	59279
chr18	47850235	47850854	SMAD2	4.1338	4.41116	intron (NM_001135937, intron 6 of 9)	80055
chr13	96463654	96463887	MIR4501	4.03501	2.78544	intron (NM_153456, intron 1 of 1)	36541
chr7	154433854	154434134	DPP6	4.03501	2.78544	intron (NM_001936, intron 1 of 25)	128782
chr8	53788165	53788399	ATP6V1H	4.03501	2.78544	intron (NM_015941, intron 9 of 13)	54760
chr1	225600135	225600401	ENAH	3.98207	3.16711	intron (NM_018212, intron 1 of 13)	52875
chr3	114973072	114973302	ZBTB20	3.92826	2.63478	intron (NM_001164343, intron 2 of 11)	-72565
chr17	1062672	1064814	ABR	3.89171	6.93634	intron (NM_001322840, intron 10 of 22).2	15403
chr17	80930218	80930497	CHMP6	3.85139	4.36003	intron (NM_020761, intron 24 of 33)	-61484
chr5	66171406	66171722	SREK1	3.83191	2.49822	intron (NM_001323527, intron 10 of 12)	26729
chr11	84558005	84558258	DLG2	3.76268	2.39177	intron (NM_001142699, intron 6 of 27)	-240792
chr19	55207710	55208115	PTPRH	3.69876	2.08928	intron (NM_001161440, intron 1 of 17)	1594
chr7	82924539	82924864	PCLO	3.69876	2.08928	intron (NM_014510, intron 6 of 19)	238180
chr5	24633773	24634001	CDH10	3.69876	2.08928	intron (NM_001317222, intron 1 of 10)	11091
chr15	34905064	34905383	AQR	3.69876	2.08928	intron (NM_014691, intron 18 of 34)	64571
chr7	22191257	22191499	RAPGEF5	3.69773	2.50103	intron (NM_012294, intron 11 of 25)	165536
chr1	198292108	198292350	NEK7	3.64676	2.03425	intron (NM_133494, intron 7 of 9)	135251
chr21	46293631	46294157	YBEY	3.63148	2.19052	intron (NM_001314023, intron 3 of 4)	7564
chr3	185454262	185455266	TMEM41A	3.63148	2.19052	intron (NM_004721, intron 7 of 13)	44293
chr7	158915413	158916677	WDR60	3.50254	5.14244	intron (NM_018051, intron 14 of 24)	59467
chr2	44391282	44391590	CAMKMT	3.48953	1.99468	intron (NM_024766, intron 3 of 10)	29532
chr3	78854582	78854842	ROBO1	3.44484	2.4994	intron (NM_133631, intron 2 of 28)	164747
chr2	204634661	204634908	PARD3B	3.4132	1.88164	intron (NM_205863, intron 1 of 21)	88991
chr7	74844849	74845146	GTF2IRD2	3.4132	1.88164	intron (NM_001281447, intron 1 of 2)	6579
chr15	27307121	27307367	GABRG3-AS1	3.36251	1.4489	intron (NM_033223, intron 3 of 9)	-145925
chr15	68528322	68528561	CORO2B	3.36251	1.4489	intron (NM_001324014, intron 1 of 12)	10068
chr4	91239960	91240346	CCSER1	3.36251	1.4489	intron (NM_001145065, intron 10 of 10)	1005122
chr22	45328055	45328315	FAM118A	3.32886	1.57539	intron (NM_001104595, intron 5 of 9)	18281
chr17	50085935	50086184	PDK2	3.27368	2.5708	intron (NM_002204, intron 23 of 25)	-8678
chr1	216030272	216030514	LOC102723833	3.25324	1.46441	intron (NM_206933, intron 32 of 71)	-42072
chrX	298023	298299	PLCXD1	3.12877	1.3209	intron (NR_028057, intron 7 of 8).2	16767
chr9	137327703	137328459	NRARP	2.55564	16.51973	intron (NM_017820, intron 17 of 21)	-25830
chr6	157310957	157314153	TMEM242	2.36809	4.98456	intron (NM_018452, intron 3 of 3)	11704
chr17	80744074	80744464	LOC101928855	2.28891	2.27604	intron (NM_020761, intron 5 of 33)	61363
chr7	905830	906135	ADAP1	2.25978	1.40885	intron (NM_001284308, intron 4 of 10)	14907

Supplementary Table 4. List of ALYREF target genes identified from ALYREF ChIP-seq experiment, related to Fig. 5

Fold enrichment stratification of 57 ALYREF target genes identified from ALYREF ChIPSeq experiment in SK-N-BE(2)C cells.

PRIMER SEQUENCES	SOURCE	IDENTIFIER
Primer sequences for gene expression analysis		
MYCN forward (F) 5'-CGACCACAAGGCCCTCAGTA-3'<br><br>MYCN reverse (R) 5'-CAGCCTTGGTGTTGGAGGAG-3'	This paper	N/A
cMYC forward (F) 5'-CGTCTCCACACATCAGCACAA-3'<br><br>cMYC reverse (R) 5'-TCTTGGCAGCAGGATAGTCCTT-3'	This paper	N/A
ALYREF forward (F) 5'-CTCCCAGGAACTCTTTGCTGAA-3'<br><br>ALYREF reverse (R) 5'-CTGCCTTCCGCTCAAAGTG-3'	This paper	N/A
Beta-2-Microglobulin (β2M) forward (F)<br><br>5'-ACTGGTCTTTCTATCTCTTGTACTACACTGA-3',<br><br>Beta-2-Microglobulin (β2M) reverse (R)<br><br>5'-TGATGCTGCTTACATGTCTCGAT-3'	This paper	N/A
USP3 forward (F) 5'-TAGAAGCGACACCAGACGGA-3'<br><br>USP3 reverse (R) 5'-GCATGGCCATTCACATACCTTC-3'	This paper	N/A
Primer sequences for chromatin immunoprecipitation	This paper	N/A
NEGATIVE CONTROL forward (F) 5'-ACATGGGTACCAACCACCTG-3'<br><br>NEGATIVE CONTROL reverse (R) 5'-AGGCTGGTCTCGAACTGTCA-3'	This paper	N/A
ALYREF forward (F) 5'-GTAGGGCGGTGCGTGATTAG-3'<br><br>ALYREF reverse (R) 5'-TAGGCTCCGCCTCCAACG-3'	This paper	N/A
USP3 forward (F) 5'-ACACTGTTCAGACTTTTAGCTTGC-3'<br><br>USP3 reverse (R) 5'-GGGGGTAACAAAAGACCAAATTTTA-3'	This paper	N/A
Primer sequences for chromatin conformation capture assay	This paper	N/A
PCR Amplicon#1 forward (F) 5'-TTTATAGTGTTTGCAGCTCAGTG-3'<br><br>PCR Amplicon#1 reverse (R) 5'-GCTACACACTAAATTACTCACTGGT-3'	This paper	N/A
PCR Amplicon#2 forward (F) 5'-GGCTGGACAGTTTTTATCGTG-3'<br><br>PCR Amplicon#2 reverse (R) 5'-GCTACACACTAAATTACTCACTGGT-3'	This paper	N/A

Supplementary Table 5. List of primer sequences used.
