# Supplementary material for: An ALYREF-MYCN coactivator complex drives neuroblastoma tumorigenesis through effects on USP3 and MYCN stability
Source: Nat Commun. 2021 Mar 25;12:1881. doi: 10.1038/s41467-021-22143-x (PMC7994381; doi:10.1038/s41467-021-22143-x)
Supplement: Supplementary file 3 — Description of Additional Supplementary Files [file 41467_2021_22143_MOESM3_ESM.pdf]

Description of Additional Supplementary Files

File Name: Supplementary Data 1

Description: **List of genes differently expressed on 17q21-ter gain vs diploid 17q21-ter locus, related to Fig. 1**

DESeq2 (differential gene expression) output for those genes that were significantly more highly expressed in patients with 17q21-ter gain on the 17q21-ter locus in the TARGET neuroblastoma patient cohort. Two-sided Wald Chi-Squared tests between patient groups were first performed to generate p-values, followed by use of the Benjamini-Hochberg method to adjust p-values for multiple comparisons (DeSeq2 methodology).

File Name: Supplementary Data 2

Description: **Correlation analysis of genes located on 17q21-ter locus with gene expression and copy number, related to Fig.1**

Results of Pearson correlations for those genes on the 17q21-ter locus that were both significantly and positively correlated in regard to their gene expression and copy number. Parametric Pearson correlation tests were conducted between each gene and MYCN to derive correlation coefficients (r). T-statistics were then calculated (where degrees of freedom = 152) from which p-values were generated from, via null hypothesis testing (two-sided). Confidence intervals, effect sizes (n), degrees of freedom and p-values are provided. Finally, p-values were adjusted for multiple comparisons using the Benjamini-Hochberg method.
