# Supplementary material for: An ALYREF-MYCN coactivator complex drives neuroblastoma tumorigenesis through effects on USP3 and MYCN stability
Source: Nat Commun. 2021 Mar 25;12:1881. doi: 10.1038/s41467-021-22143-x (PMC7994381; doi:10.1038/s41467-021-22143-x)
Supplement: Supplementary file 6 — Reporting Summary [file 41467_2021_22143_MOESM6_ESM.pdf]

nature research

Corresponding author(s): Glenn Marshall and Belamy Cheung

Last updated by author(s): Jan 26, 2021

Reporting Summary

Nature Research wishes to improve the reproducibility of the work that we publish. This form provides structure for consistency and transparency in reporting. For further information on Nature Research policies, see Authors & Referees and the Editorial Policy Checklist.

Statistics

For all statistical analyses, confirm that the following items are present in the figure legend, table legend, main text, or Methods section.

n/a	Confirmed	
☐	☒	The exact sample size (*n*) for each experimental group/condition, given as a discrete number and unit of measurement
☐	☒	A statement on whether measurements were taken from distinct samples or whether the same sample was measured repeatedly
☐	☒	The statistical test(s) used AND whether they are one- or two-sided<br>*Only common tests should be described solely by name; describe more complex techniques in the Methods section.*
☐	☒	A description of all covariates tested
☐	☒	A description of any assumptions or corrections, such as tests of normality and adjustment for multiple comparisons
☐	☒	A full description of the statistical parameters including central tendency (e.g. means) or other basic estimates (e.g. regression coefficient) AND variation (e.g. standard deviation) or associated estimates of uncertainty (e.g. confidence intervals)
☐	☒	For null hypothesis testing, the test statistic (e.g. *F*, *t*, *r*) with confidence intervals, effect sizes, degrees of freedom and *P* value noted<br>*Give P values as exact values whenever suitable.*
☒	☐	For Bayesian analysis, information on the choice of priors and Markov chain Monte Carlo settings
☒	☐	For hierarchical and complex designs, identification of the appropriate level for tests and full reporting of outcomes
☐	☒	Estimates of effect sizes (e.g. Cohen's *d*, Pearson's *r*), indicating how they were calculated

Our web collection on statistics for biologists contains articles on many of the points above.

Software and code

Policy information about availability of computer code

Data collection	No custom algorithms or software was used for data collection. All other software are described and cited in the manuscript. Whole genome sequencing (WGS) data were obtained through the TARGET data matrix (https://ocg.cancer.gov/programs/target/data-matrix) and further processed using the R statistical language and RStudio (1.1.456). We utilised public data resources produced by the Cancer Cell Line Encyclopedia (CCLE) and Project Achilles via the Cancer Dependency Map portal (DepMap, 20Q1). Gene expression, copy number data were first obtained from DepMap and then filtered using R/R Studio (1.1.456).
Data analysis	No custom algorithms or software were used for data analysis. All other software are described and cited in the manuscript.<br>For ChIPSeq analysis, reads from fastq files were first quality trimmed using TrimGalore (v0.4.5), then aligned to the human reference genome (GRCh38) using bowtie2 (v2.1.0), resulting sam files were converted to bam files and sorted using samtools (v1.9). Reads aligned to ENCODE blacklisted regions were removed using bedtools (v2.27.1). Peaks were then called using MACS2 (v2.1.1). Fold enrichment tracks were also generated using MACS2 (v2.1.1) and converted to the bigwig format using bedtools (v2.27.1). HOMER (v4.10.3) was used to annotate peaks. GRCh38 annotated peak text file produced from MACS2 (v2.1.1) and then annotated using HOMER (v4.10.3) bigWig file generated by converting MACS2 (v2.1.1) fold enrichment bedgraph file using bedtools (v2.27.1). The TH-MYCN +/+ mice microarray data were analysed in R [http://www.r-project.org/] and normalized using GenePattern software (version 3.2.3 Broad Institute) with the AgilentToGCT and LimmaGP modules (version 19.3) available at https://pwbc.garvan.unsw.edu.au/gp. Average ploidy values from Whole genome sequencing (WGS) data were calculated in 10 kilobase bins across chromosome 17 using the Genomic Ranges R package (v1.40.0). We utilised matched clinical annotations containing event-free and overall survival data to construct Kaplan-Meier survival curves of the different molecular subgroups using the survminer (v0.4.6) and survival R packages (v2.42.1). We performed differential gene expression testing between 17q21-ter gain vs diploid as well as MYCN- amplified vs non-amplified samples using the DESeq2 R package (v1.22.0). We used the survival R package (v2.42.1) for univariate Cox proportional-hazards (CoxPH) regression models.

For manuscripts utilizing custom algorithms or software that are central to the research but not yet described in published literature, software must be made available to editors/reviewers. We strongly encourage code deposition in a community repository (e.g. GitHub). See the Nature Research guidelines for submitting code & software for further information.

Data

Policy information about availability of data

All manuscripts must include a data availability statement. This statement should provide the following information, where applicable:

- Accession codes, unique identifiers, or web links for publicly available datasets
- A list of figures that have associated raw data
- A description of any restrictions on data availability

The ALYREF ChIP-seq data has been deposited at the Gene Expression Omnibus Website with series number of GSE150303 (https://www.ncbi.nlm.nih.gov/geo/query/acc.cgi?acc=GSE150303). We also obtained several other publicly available ChIP-seq datasets (GSE80151) of MYCN, RNA Polymerase II, BRD4, H3K27ac, H3K4me3 and H3K27me3 to complement our ALYREF ChIP-seq data (GSE80151)(https://www.ncbi.nlm.nih.gov/geo/query/acc.cgi?acc=GSE80151), fastq files were obtained directly from the European Nucleotide Archive (ENA) under the study accession PRJNA318044 (https://www.ncbi.nlm.nih.gov/bioproject/?term=PRJNA318044). Gene expression and relevant patient prognosis information in TARGET, SEQC and Kocak neuroblastoma patient datasets were downloaded from R2 platform (http://r2.amc.nl). Whole genome sequencing (WGS) data were obtained through the TARGET data matrix (https://ocg.cancer.gov/programs/target/data-matrix). RNA-seq data which had paired WGS data, were also obtained from the TARGET data matrix. RNA-seq data for the SEQC neuroblastoma cohort were obtained from the gene expression omnibus (GEO) with the accession GSE62564 (https://www.ncbi.nlm.nih.gov/geo/query/acc.cgi?acc=GSE62564). We utilised public data resources produced by the Cancer Cell Line Encyclopedia (CCLE) and Project Achilles via the Cancer Dependency Map (DepMap, 20Q1) portal (https://depmap.org/portal/). Uncropped and unprocessed immunoblot scans as well as colony formation assay and PCR agarose gel pictures for all main figures are provided as Supplementary Information. All other relevant data are available from the corresponding authors on request.

Field-specific reporting

Please select the one below that is the best fit for your research. If you are not sure, read the appropriate sections before making your selection.

☒ Life sciences ☐ Behavioural & social sciences ☐ Ecological, evolutionary & environmental sciences

For a reference copy of the document with all sections, see nature.com/documents/nr-reporting-summary-flat.pdf

Life sciences study design

All studies must disclose on these points even when the disclosure is negative.

Sample size	For in vitro experiments, three biological replicates were chosen as minimum sample size. For rescue ubiquitin experiments, sample size was chosen to be as large as possible whilst producing datasets of a practical size for use in subsequent analysis. For in vivo experiments we used standard sample sizes based on preliminary experiments and sample sizes reported in the literature previously. The numbers of performed independent experiments were indicated in each figure and figure legend.<br>Animal sample sizes were estimated according to our previous work (Ooi et al., Can Res, 2018, doi:10.1158/0008-5472) in which we carried out similar studies for neuroblastoma progression. MYCN ubiquitination sample sizes were estimated according to previous published work (Tavana et al., Nat Med, 2016, doi:10.1038/nm.4180).
Data exclusions	Animals were only excluded from analyses when they were excluded from the experiments, because they did not develop tumors after xenografting with neuroblastoma cells.
Replication	All in vitro experiments (except for ChIP-seq and rescue MYCN ubiquitination) were performed at least 3 times and we were able to confirm the reproducibility of our results. All attempts at replication were successful and are included in the data analyses. All experiments were independently repeated to ensure the findings are reproducible. For cellular and molecular experiments, each single measurement was performed at least in triplicate and the results were consistently reproducible. In vivo experiments were performed in 8 mice per experimental group.
Randomization	All mice for in vivo data and cells for in vitro experiments were randomly assigned to experimental and treatment groups.
Blinding	Investigators were not blinded during gene expression analysis in human tumor tissues, to complete analyses correctly, sample information and corresponding clinical data were known to investigators. Investigators were not blinded during in vivo studies, as the doxycycline treatment was given by cage.

Reporting for specific materials, systems and methods

We require information from authors about some types of materials, experimental systems and methods used in many studies. Here, indicate whether each material, system or method listed is relevant to your study. If you are not sure if a list item applies to your research, read the appropriate section before selecting a response.

Materials & experimental systems

n/a	Involved in the study
☐	☒ Antibodies
☐	☒ Eukaryotic cell lines
☒	☐ Palaeontology
☐	☒ Animals and other organisms
☒	☐ Human research participants
☒	☐ Clinical data

Methods

n/a	Involved in the study
☐	☒ ChIP-seq
☒	☐ Flow cytometry
☒	☐ MRI-based neuroimaging

Antibodies

Antibodies used

Mouse monoclonal anti-MYCN antibody for immunoprecipitation, Merck Millipore, Cat# OP13
Rabbit monoclonal anti-ALYREF (D3R4R) antibody for immunoblot and immunoprecipitation, Cell Signaling, Cat# 12655S, Lot#1
Mouse monoclonal anti-DYKDDDDK tag (9A3) antibody for immunoblotting and immunoprecipitation, Cell Signaling, Cat# 8146S, Lot#3
Rat monoclonal anti-HA (3F10) antibody for immunoblotting, Sigma-Aldrich, Cat# 11867431001
Mouse monoclonal anti-MYCN (B84B) antibody for immunoblotting and ChIP, Santa Cruz Biotechnology, Cat#: sc-53993, Lot#J2918, RRID:AB_831602
Rabbit monoclonal anti-cMYC (D84C12) antibody for immunoblotting, Cell Signaling, Cat# 5605, Lot#11
Rabbit monoclonal linkage-specific (K-63) anti-ubiquitin (HWA4C4) antibody for immunoblotting, Invitrogen, Cat# 14607782, Lot#2097179
Rabbit monoclonal linkage-specific (K-48) anti-ubiquitin (EP8589) antibody for immunoblotting, Abcam, Cat# 140601, Lot#GR298739-14
Mouse monoclonal anti-ubiquitin (P4D1) antibody for immunoblotting, Cell Signaling, Cat# 3936
Rabbit polyclonal Topoisomerase I antibody for immunoblotting, Novus Biologicals,Cat# NBP1-30481
Rabbit polyclonal anti-USP3 antibody for immunoblotting, Invitrogen, Cat# PA5-85512, Lot# UF2794327C
Mouse anti-ALYREF (11G5) antibody for ChIP and ChIPSeq, ImmunoQuest Ltd. Cat# IQ221
Mouse monoclonal anti-β-actin (AC-15) antibody for immunoblotting, Sigma Aldrich, Cat#: A1978
Mouse monoclonal anti-GAPDH (G-9) antibody for immunoblotting, Santa Cruz Biotechnology, Cat# sc-365062, Lot#C2119
Mouse anti-BrdU for BrdU assay, part of BrdU proliferation kit by Roche, Sigma-Aldrich, Cat# 11647229001
Anti-mouse IgG-Alexa-Flour-594 for BrdU assay, part of BrdU proliferation kit by Roche, Sigma-Aldrich, Cat# 11647229001
Rabbit control IgG antibody for immunoprecipitation, Santa Cruz Biotechnology, Cat# sc-2027
Mouse control IgG antibody for immunoprecipitation, Santa Cruz Biotechnology, Cat# sc-2025
Goat anti-rabbit IgG-HRP for immunoblotting, Santa Cruz Biotechnology, Cat # sc-2004
Goat anti-mouse IgG-HRP for immunoblotting, Santa Cruz Biotechnology, Cat # sc-2005
Goat Anti-Rat IgG-HPR for immunoblotting, Merck Millipore, Cat # DC01L

Validation

All primary antibodies were validated previously by manufacturer and published papers at the manufacturer's website. We also validated all antibodies in our own experiments for both endogenous and exogenous expressions (when applicable) with proper molecular weight markers and positive/negative controls. Series of antibody dilutions were tested and optimized dilutions are provided in the Methods section. When dilution is not applicable (immunoprecipitation and ChIP experiments), antibody concentrations are provided in the Methods section.

Mouse monoclonal anti-MYCN antibody for immunoprecipitation, Merck Millipore, Cat# OP13
Manufacturer website: https://www.sigmaaldrich.com/catalog/product/mm/op13?lang=en&region=AU
All citations:
1. Glycine decarboxylase is a transcriptional target of MYCN required for neuroblastoma cell proliferation and tumorigenicity. Ahmet Alptekin et. al Oncogene, 38(50), 7504-7520 (2019-8-25)

Rabbit monoclonal anti-ALYREF (D3R4R) antibody for immunoblot and immunoprecipitation, Cell Signaling, Cat# 12655S, Lot#1
Manufacturer website: https://www.cellsignal.com/products/primary-antibodies/thoc4-aly-d3r4r-rabbit-mab/12655
All citations:
1.Journal: PLoS One Applications: Western Blotting (WB) Reactivity: Unspecified Title: Genome-wide analysis of HOXC4 and HOXC6 regulated genes and binding sites in prostate cancer cells. Author: Zhifei Luo, et. al. Year: 2020
2.Journal: Cell Rep Applications: Unspecified Reactivity: Unspecified Title: A Cell-Line-Specific Atlas of PARP-Mediated Protein Asp/Glu-ADP-Ribosylation in Breast Cancer. Author: Yuanli Zhen, et. al. Year: 2017

Mouse monoclonal anti-DYKDDDDK tag (9A3) antibody for immunoblotting and immunoprecipitation, Cell Signaling, Cat# 8146S, Lot#3
Manufacturer website: https://www.cellsignal.com/products/wb-ip-reagents/dykddddk-tag-9a3-mouse-mab-binds-to-same-epitope-as-sigma-s-anti-flag-m2-antibody-sepharose-bead-conjugate/575
top 10 selected citations for western blotting and immunoprecipitation:

Journal:
Mol Med Rep
Applications:
Western Blotting (WB)
Reactivity:
Unspecified

Title:
Septin4 regulates endoplasmic reticulum stress and apoptosis in melatonin-induced osteoblasts.
Author:
Lin Tao, et. al.
Year:
2020

Journal:
Nat Commun
Applications:
Western Blotting (WB)
Reactivity:
Unspecified
Title:
Long noncoding RNA AGPG regulates PFKFB3-mediated tumor glycolytic reprogramming.
Author:
Jia Liu, et. al.
Year:
2020

Journal:
Nat Commun
Applications:
Western Blotting (WB)
Reactivity:
Unspecified
Title:
YAP1/TAZ-TEAD transcriptional networks maintain skin homeostasis by regulating cell proliferation and limiting KLF4 acti...
Author:
Yao Yuan, et. al.
Year:
2020

Journal:
Int J Oncol
Applications:
Immunoprecipitation (IP), Western Blotting (WB)
Reactivity:
Unspecified
Title:
Interaction of YAP1 and mTOR promotes bladder cancer progression.
Author:
Mingxi Xu, et. al.
Year:
2020

Journal:
PLoS Pathog
Applications:
Western Blotting (WB)
Reactivity:
Unspecified
Title:
KSHV activates unfolded protein response sensors but suppresses downstream transcriptional responses to support lytic re...
Author:
Benjamin P Johnston, et. al.
Year:
2019

Journal:
Nat Commun
Applications:
Western Blotting (WB)
Reactivity:
Unspecified
Title:
Glycogen branching enzyme controls cellular iron homeostasis via Iron Regulatory Protein 1 and mitoNEET.
Author:
Nhan Huynh, et. al.
Year:
2019

Journal:
Nat Commun

Applications:
Western Blotting (WB)
Reactivity:
Unspecified
Title:
N6-methyladenosine modification of circNSUN2 facilitates cytoplasmic export and stabilizes HMGA2 to promote colorectal l...
Author:
Ri-Xin Chen, et. al.
Year:
2019

Journal:
Cell Death Dis
Applications:
Western Blotting (WB)
Reactivity:
Unspecified
Title:
Dominant-negative ATF5 rapidly depletes survivin in tumor cells.
Author:
Xiaotian Sun, et. al.
Year:
2019

Journal:
Cell Death Dis
Applications:
Immunoprecipitation (IP)
Reactivity:
Homo sapiens (Human)
Title:
CCT3 acts upstream of YAP and TFCP2 as a potential target and tumour biomarker in liver cancer.
Author:
Ya Liu, et. al.
Year:
2019

Journal:
Nat Commun
Applications:
Western Blotting (WB)
Reactivity:
Homo sapiens (Human)
Title:
Brain somatic mutations observed in Alzheimer's disease associated with aging and dysregulation of tau phosphorylation.
Author:
Jun Sung Park, et. al.
Year
2019

Journal:
Redox Biol
Applications:
Western Blotting (WB)
Reactivity:
Homo sapiens (Human)
Title
Ferroptosis is governed by differential regulation of transcription in liver cancer.
Author:
Xiao Zhang, et. al
Year:
2019

Rat monoclonal anti-HA (3F10) antibody for immunoblotting, Sigma-Aldrich, Cat# 11867431001
Manufacturer website:https://www.sigmaaldrich.com/catalog/product/roche/roahaha?lang=en&region=AU&cm_sp=Insite-_-caContent_prodMerch_gruCrossEntropy-_-prodMerch1
All citations:
1. A COFRADIC protocol to study protein ubiquitination.
Elisabeth Stes et. al
Journal of proteome research, 13(6), 3107-3113 (2014-5-13)
2. Hypoxia interferes with connective tissue growth factor (CTGF) gene expression in human proximal tubular cell lines.
Sven Kroening et. al
Nephrology, dialysis, transplantation : official publication of the European Dialysis and Transplant Association - European Renal Association, 24(11), 3319-3325 (2009-6-25)
3. Amino Acid-Dependent mTORC1 Regulation by the Lysosomal Membrane Protein SLC38A9.

Jennifer Jung et. al
Molecular and cellular biology, 35(14), 2479-2494 (2015-5-13)
4. Comprehensive substrate specificity profiling of the human Nek kinome reveals unexpected signaling outputs.
Bert van de Kooij et. al
eLife, 8, undefined (2019-5-28)
5. Structures of PGAM5 Provide Insight into Active Site Plasticity and Multimeric Assembly.
Apirat Chaikuad et. al
Structure (London, England : 1993), 25(7), 1089-1099 (2017-6-27)
6. The mitochondrial DNA polymerase gamma degrades linear DNA fragments precluding the formation of deletions.
Nadee Nissanka et. al
Nature communications, 9(1), 2491 (2018-6-29)

Mouse monoclonal anti-MYCN (B84B) antibody for immunoblotting and ChIP, Santa Cruz Biotechnology, Cat#: sc-53993, Lot#J2918, RRID:AB_831602
Manufacturer website: https://www.scbt.com/p/n-myc-antibody-b8-4-b
All citations:
1. Identification and Pharmacological Inactivation of the MYCN Gene Network as a Therapeutic Strategy for Neuroblastic Tumor Cells*The Journal of Biological ChemistryDecember 4, 2014 Olesya Chayka, Cosimo Walter D'Acunto, Odette Middleton, Maryam Arab, Arturo Sala
2. Inactivation of SMC2 shows a synergistic lethal response in MYCN-amplified neuroblastoma cellsCell CycleFebruary 7, 2014Yuko Murakami-Tonami, Satoshi Kishida, Ichiro Takeuchi, Yuki Katou, John M Maris, Hitoshi Ichikawa, Yutaka Kondo, Yoshitaka Sekido, Katsuhiko Shirahige, Hiroshi Murakami, Kenji Kadomatsu

Rabbit monoclonal anti-cMYC (D84C12) antibody for immunoblotting, Cell Signaling, Cat# 5605, Lot#11
Manufacturer website: https://www.cellsignal.com/products/primary-antibodies/c-myc-d84c12-rabbit-mab/5605?site-search-type=Products&N=4294956287&Ntt=c-myc+antibody+%235602&fromPage=plp
All citations:
Journal: Cancers (Basel) Applications:
Immunocytochemistry (ICC), Western Blotting
Reactivity:
Homo sapiens (Human)
Title: NGF-Enhanced Vasculogenic Properties of Epithelial Ovarian Cancer Cells Is Reduced by Inhibition of the COX-2/PGE2 Signa...
Author: Maritza P Garrido, et. al.
Year: 2019

Journal: Nat Commun Applications:
Western Blotting (WB)
Reactivity:
Homo sapiens (Human)
Title: Prolyl hydroxylase substrate adenylosuccinate lyase is an oncogenic driver in triple negative breast cancer.
Author: Giada Zurlo, et. al.
Year: 2019

Journal: Molecules Applications:
Western Blotting (WB)
Reactivity:
Homo sapiens (Human)
Title: Synthesis of 9-Hydroxystearic Acid Derivatives and Their Antiproliferative Activity on HT 29 Cancer Cells.
Author: Natalia Calonghi, et. al.
Year: 2019

Journal: Ther Adv Med Oncol Applications:
Western Blotting (WB)
Reactivity:
Homo sapiens (Human)
Title: High blood sugar levels but not diabetes mellitus significantly enhance oxaliplatin chemoresistance in patients with sta...
Author: I-Ping Yang, et. al.
Year: 2019

Journal: Nat Commun Applications:
Western Blotting (WB)
Reactivity:
Homo sapiens (Human)
Title: GREB1 induced by Wnt signaling promotes development of hepatoblastoma by suppressing TGFβ signaling.
Author: Shinji Matsumoto, et. al.
Year: 2019

Journal: Cell Commun Signal Applications:
Western Blotting (WB)
Reactivity:
Homo sapiens (Human)
Title: WNT5B governs the phenotype of basal-like breast cancer by activating WNT signaling.
Author: Shaojie Jiang, et. al.
Year: 2019

Journal: Oncol Lett Applications:
Western Blotting (WB)
Reactivity:
Homo sapiens (Human)
Title: lncRNA HOTTIP facilitates osteosarcoma cell migration, invasion and epithelial-mesenchymal transition by forming a posit...
Author: Yang Tang, et. al.
Year: 2019

Journal: Oncol Rep Applications:
Western Blotting (WB)
Reactivity:
Homo sapiens (Human)
Title: Effects of LDOC1 on colorectal cancer cells via downregulation of the Wnt/β-catenin signaling pathway.
Author: Jiayi Jiang, et. al.
Year: 2019

Journal: Mol Med Rep Applications:
Western Blotting (WB)
Reactivity:
Homo sapiens (Human)
Title: Effect of miR 145 on gastric cancer cells.
Author: Jia Wang, et. al.
Year: 2019

Journal: Cell Death Dis Applications:
Western Blotting (WB)
Reactivity:
Homo sapiens (Human)
Title: Regulation of senescence escape by TSP1 and CD47 following chemotherapy treatment.
Author: Jordan Guillon, et. al.
Year: 2019

Journal: Int J Mol Sci Applications:
Western Blotting (WB)
Reactivity:
Homo sapiens (Human)
Title: Interaction between Tumor-Associated Dendritic Cells and Colon Cancer Cells Contributes to Tumor Progression via CXCL1.
Author: Ya-Ling Hsu, et. al.
Year: 2018

Journal: Mol Med Rep Applications:
Western Blotting (WB)
Reactivity:
Homo sapiens (Human)
Title: Matrine inhibits prostate cancer via activation of the unfolded protein response/endoplasmic reticulum stress signaling ...
Author: Junli Chang, et. al.
Year: 2018

Journal: J Gerontol A Biol Sci Med Sci Applications:
Western Blotting (WB)
Reactivity:
Homo sapiens (Human)
Title: Short-term Low-Dose mTORC1 Inhibition in Aged Rats Counter-Regulates Age-Related Gene Changes and Blocks Age-Related Kid...
Author: Tea Shavlakadze, et. al.
Year: 2018

Journal: Oncol Lett Applications:
Western Blotting (WB)
Reactivity:
Homo sapiens (Human)
Title: Suppression of tumor cell proliferation and migration by human umbilical cord mesenchymal stem cells: A possible role fo...
Author: Yin Yuan, et. al.
Year: 2018

Journal: EMBO Mol Med Applications:
Western Blotting (WB)
Reactivity:
Homo sapiens (Human)
Title: Anti-tumor efficacy of a novel CLK inhibitor via targeting RNA splicing and MYC-dependent vulnerability.
Author: Kenichi Iwai, et. al.
Year: 2018

Journal: Oncotarget Applications:

Western Blotting (WB)
Reactivity:
Homo sapiens (Human)
Title: Bromodomain protein BRD4 inhibitor JQ1 regulates potential prognostic molecules in advanced renal cell carcinoma.
Author: Takashi Sakaguchi, et. al.
Year: 2018

Journal: Front Pharmacol Applications:
Western Blotting (WB)
Reactivity:
Homo sapiens (Human)
Title: 1,2,3,4,6-Penta-O-Galloyl-Beta-D-Glucopyranoside Inhibits Proliferation of Multiple Myeloma Cells Accompanied with Suppr...
Author: Duurenjargal Tseeleesuren, et. al.
Year: 2018

Journal: Oncotarget Applications:
Western Blotting (WB)
Reactivity:
Homo sapiens (Human)
Title: Lactate-activated macrophages induced aerobic glycolysis and epithelial-mesenchymal transition in breast cancer by regul...
Author: Sensen Lin, et. al.
Year: 2017

Journal: Nat Med Applications:
Western Blotting (WB)
Reactivity:
Homo sapiens (Human)
Title: The N6-methyladenosine (m6A)-forming enzyme METTL3 controls myeloid differentiation of normal hematopoietic and leukemia...
Author: Ly P Vu, et. al.
Year: 2017

Journal: Cancers (Basel) Applications:
Western Blotting (WB)
Reactivity:
Homo sapiens (Human)
Title: STAT3 but Not HIF-1α Is Important in Mediating Hypoxia-Induced Chemoresistance in MDA-MB-231, a Triple Negative Breast C...
Author: Hoda Soleymani Abyaneh, et. al.
Year: 2017

Journal: Sci Rep Applications:
Western Blotting (WB)
Reactivity:
Homo sapiens (Human)
Title: Hypoxic Stress Decreases c-Myc Protein Stability in Cardiac Progenitor Cells Inducing Quiescence and Compromising Their ...
Author: Michael A Bellio, et. al.
Year: 2017

Journal: Oncogene Applications:
Western Blotting (WB)
Reactivity:
Homo sapiens (Human)
Title: SPOP regulates prostate epithelial cell proliferation and promotes ubiquitination and turnover of c-MYC oncoprotein.
Author: C Geng, et. al.
Year: 2017

Journal: Cell Death Dis Applications:
Western Blotting (WB)
Reactivity:
Homo sapiens (Human)
Title: An autocrine inflammatory forward-feedback loop after chemotherapy withdrawal facilitates the repopulation of drug-resis...
Author: Deyong Jia, et. al.
Year: 2017

Journal: Sci Rep Applications:
Immunohistochemistry (IHC), Western Blotting ...
Reactivity:
Homo sapiens (Human)
Title: Ribosomal protein L23 negatively regulates cellular apoptosis via the RPL23/Miz-1/c-Myc circuit in higher-risk myelodysp...
Author: Yuekun Qi, et. al.

Year: 2017

Journal: Sci Rep Applications:
Western Blotting (WB)
Reactivity:
Homo sapiens (Human)
Title: Secreted Frizzled-related protein 4 (sFRP4) chemo-sensitizes cancer stem cells derived from human breast, prostate, and ...
Author: A Deshmukh, et. al.
Year: 2017

Journal: J Exp Clin Cancer Res Applications:
Western Blotting (WB)
Reactivity:
Homo sapiens (Human)
Title: CIP2A mediates fibronectin-induced bladder cancer cell proliferation by stabilizing β-catenin.
Author: Fengbin Gao, et. al.
Year: 2017

Journal: Mol Cell Applications:
Western Blotting (WB)
Reactivity:
Homo sapiens (Human)
Title: Bromodomain Protein BRD4 Is a Transcriptional Repressor of Autophagy and Lysosomal Function.
Author: Jun-Ichi Sakamaki, et. al.
Year: 2017

Journal: Cell Death Dis Applications:
Western Blotting (WB)
Reactivity:
Homo sapiens (Human)
Title: Induction of intestinal stemness and tumorigenicity by aberrant internalization of commensal non-pathogenic E. coli.
Author: Upasana Sahu, et. al.
Year: 2017

Journal: Nat Commun Applications:
Western Blotting (WB)
Reactivity:
Homo sapiens (Human)
Title: The OncoPPi network of cancer-focused protein-protein interactions to inform biological insights and therapeutic strateg...
Author: Zenggang Li, et. al.
Year: 2017

Journal: Mol Med Rep Applications:
Western Blotting (WB)
Reactivity:
Homo sapiens (Human)
Title: NLS RARα modulates acute promyelocytic leukemia NB4 cell proliferation and differentiation via the PI3K/AKT pathway.
Author: Hao Song, et. al.
Year: 2016

Journal: Nat Cell Biol Applications:
Western Blotting (WB)
Reactivity:
Homo sapiens (Human)
Title: Induction of LIFR confers a dormancy phenotype in breast cancer cells disseminated to the bone marrow.
Author: Rachelle W Johnson, et. al.
Year: 2016

Journal: Cell Death Discov Applications:
Western Blotting (WB)
Reactivity:
Homo sapiens (Human)
Title: Mitochondrial oligomers boost glycolysis in cancer stem cells to facilitate blebbishield-mediated transformation after a...
Author: G G Jinesh, et. al.
Year: 2016

Journal: Biomed Res Int Applications:
Western Blotting (WB)
Reactivity:
Homo sapiens (Human)
Title: Overexpression of β-Catenin Induces Cisplatin Resistance in Oral Squamous Cell Carcinoma.
Author: Long Li, et. al.
Year: 2016

Journal: Nat Commun Applications:

Western Blotting (WB)
Reactivity:
Homo sapiens (Human)
Title: The oncogenic transcription factor c-Jun regulates glutaminase expression and sensitizes cells to glutaminase-targeted t...
Author: Michael J Lukey, et. al.
Year: 2016

Journal: Exp Ther Med Applications:
Western Blotting (WB)
Reactivity:
Homo sapiens (Human)
Title: Malate dehydrogenase-2 inhibitor LW6 promotes metabolic adaptations and reduces proliferation and apoptosis in activated...
Author: Theodoros Eleftheriadis, et. al.
Year: 2015

Journal: Nat Commun Applications:
Western Blotting (WB)
Reactivity:
Homo sapiens (Human)
Title: DOT1L cooperates with the c-Myc-p300 complex to epigenetically derepress CDH1 transcription factors in breast cancer pro...
Author: Min-Hyung Cho, et. al.
Year: 2015

Journal: Cell Death Dis Applications:
Western Blotting (WB)
Reactivity:
Homo sapiens (Human)
Title: Identification of thioridazine, an antipsychotic drug, as an antiglioblastoma and anticancer stem cell agent using publi...
Author: H-W Cheng, et. al.
Year: 2015

Journal: Autophagy Applications:
Western Blotting (WB)
Reactivity:
Homo sapiens (Human)
Title: Targeting Hedgehog signaling pathway and autophagy overcomes drug resistance of BCR-ABL-positive chronic myeloid leukemi...
Author: Xian Zeng, et. al.
Year: 2015

Journal: BMC Cancer Applications:
Western Blotting (WB)
Reactivity:
Homo sapiens (Human)
Title: Identification of synthetic lethality of PRKDC in MYC-dependent human cancers by pooled shRNA screening.
Author: Zongxiang Zhou, et. al.
Year: 2014

Journal: Oncotarget Applications:
Western Blotting (WB)
Reactivity:
Homo sapiens (Human)
Title: Combination of PIM and JAK2 inhibitors synergistically suppresses MPN cell proliferation and overcomes drug resistance.
Author: Shih-Min A Huang, et. al.
Year: 2014

Journal: Mol Cell Biol Applications:
Western Blotting (WB)
Reactivity:
Homo sapiens (Human)
Title: MicroRNA 9-3p targets β1 integrin to sensitize claudin-low breast cancer cells to MEK inhibition.
Author: Jon S Zawistowski, et. al.
Year: 2013

Rabbit monoclonal linkage-specific (K-63) anti-ubiquitin (HWA4C4) antibody for immunoblotting, Invitrogen, Cat# 14607782, Lot#2097179
Manufacturer website: https://www.thermofisher.com/antibody/product/Ub-K63-Antibody-clone-HWA4C4-Monoclonal/14-6077-82
References:
1. The Journal of cell biology
The BEACH-containing protein WDR81 coordinates p62 and LC3C to promote aggrephagy.
"14-6077 was used in Western Blotting to report the essential role of the protein WDR81 in eliminating ubiquitinated proteins through autophagy."

Authors Liu X,Li Y,Wang X,Xing R,Liu K,Gan Q,Tang C,Gao Z,Jian Y,Luo S,Guo W,Yang C
Year2017
Species
Human
2. The Journal of biological chemistry
Molecular Determinants of Scaffold-induced Linear Ubiquitinylation of B Cell Lymphoma/Leukemia 10 (Bcl10) during T Cell Receptor and Oncogenic Caspase Recruitment Domain-containing Protein 11 (CARD11) Signaling.
"14-6077 was used in Western Blotting to examine the effects of Bcl10 recruitment to the CARD11 scaffold protein during T cell receptor signalling."
Authors Yang YK,Yang C,Chan W,Wang Z,Deibel KE,Pomerantz JL
Year2016
Species
Human
3. Nature communications
Fucose-specific DC-SIGN signalling directs T helper cell type-2 responses via IKKε- and CYLD-dependent Bcl3 activation.
"14-6077 was used in Western Blotting to demonstrate that recognition of fucose-expressing extracellular pathogens by DC-SIGN favours T helper cell type-2 (TH2) responses via Bcl3."
Authors Gringhuis SI,Kaptein TM,Wevers BA,Mesman AW,Geijtenbeek TB
Year2014
Species
Human
Dilution
1:250
4. Molecular and cellular biology
A quantitative signaling screen identifies CARD11 mutations in the CARD and LATCH domains that induce Bcl10 ubiquitination and human lymphoma cell survival.
"14-6077 was used in Western Blotting to identify a method of detecting CARD11 mutations with oncogeneic potential, and offer a straightforward method for the discovery of variants that promote the tumorigenesis of NF-κB-dependent lymphomas."
Authors Chan W,Schaffer TB,Pomerantz JL
Year2013
Species
Human
5. Proceedings of the National Academy of Sciences of the United States of America
Degradation of the antiviral component ARGONAUTE1 by the autophagy pathway.
"14-6077 was used in Western Blotting to investigate the mechanism of P0-mediated ARGONAUTE1 degradation using a P0-inducible transgenic Arabidopsis cell line."
Authors Derrien B,Baumberger N,Schepetilnikov M,Viotti C,De Cillia J,Ziegler-Graff V,Isono E,Schumacher K,Genschik P

Rabbit monoclonal linkage-specific (K-48) anti-ubiquitin (EP8589) antibody for immunoblotting, Abcam, Cat# 140601, Lot#GR298739-14
Manufacturer website:https://www.abcam.com/ubiquitin-linkage-specific-k48-antibody-ep8589-ab140601.html
References:
• Luo XB et al. Proinflammatory Effects of Ubiquitin-Specific Protease 5 (USP5) in Rheumatoid Arthritis Fibroblast-Like Synoviocytes. Mediators Inflamm 2020:8295149 (2020).PubMed: 32214906
• Peng H et al. The ubiquitin-specific protease USP8 directly deubiquitinates SQSTM1/p62 to suppress its autophagic activity. Autophagy 16:698-708 (2020).PubMed: 31241013
• Li Y et al. TRIM65 E3 ligase targets VCAM-1 degradation to limit LPS-induced lung inflammation. J Mol Cell Biol 12:190-201 (2020).PubMed: 31310649
• Yan K et al. A20 inhibits osteoclastogenesis via TRAF6-dependent autophagy in human periodontal ligament cells under hypoxia. Cell Prolif 53:e12778 (2020).PubMed: 32027437
• Kim SY et al. Non-Thermal Plasma Induces Antileukemic Effect Through mTOR Ubiquitination. Cells 9:N/A (2020).PubMed: 32131492
• Hu C et al. The USP10-HDAC6 axis confers cisplatin resistance in non-small cell lung cancer lacking wild-type p53. Cell Death Dis 11:328 (2020).PubMed: 32382008
• Lei Q et al. Ubiquitin C-terminal hydrolase L1 (UCHL1) regulates post-myocardial infarction cardiac fibrosis through glucose-regulated protein of 78 kDa (GRP78). Sci Rep 10:10604 (2020).PubMed: 32606430
• Meena NK et al. Enzyme Replacement Therapy Can Reverse Pathogenic Cascade in Pompe Disease. Mol Ther Methods Clin Dev 18:199-214 (2020).PubMed: 32671132
• Huang X et al. TRIM14 promotes endothelial activation via activating NF-?B signaling pathway. J Mol Cell Biol N/A:N/A (2019).PubMed: 31070748
• Yu Y et al. Inhibition of Ubiquitin-Specific Protease 14 Suppresses Cell Proliferation and Synergizes with Chemotherapeutic Agents in Neuroblastoma. Mol Cancer Ther 18:1045-1056 (2019).PubMed: 30962318
• Zeng SG et al. Hypoxia-induced internalization of connexin 26 and connexin 43 in pulmonary epithelial cells is involved in the occurrence of non-small cell lung cancer via the P53/MDM2 signaling pathway. Int J Oncol 55:845-859 (2019).PubMed: 31485592
• Zeng G et al. E3-ubiquitin ligase TRIM6 aggravates myocardial ischemia/reperfusion injury via promoting STAT1-dependent cardiomyocyte apoptosis. Aging (Albany NY) 11:3536-3550 (2019).PubMed: 31171760
• Sato K et al. Partial proteasomal degradation of Lola triggers the male-to-female switch of a dimorphic courtship circuit. Nat Commun 10:166 (2019).PubMed: 30635583
• Zhang L et al. Cysteine-rich intestinal protein 1 suppresses apoptosis and chemosensitivity to 5-fluorouracil in colorectal cancer through ubiquitin-mediated Fas degradation. J Exp Clin Cancer Res 38:120 (2019).PubMed: 30850009
• Nakayama Y et al. Identification of linear polyubiquitin chain immunoreactivity in tau pathology of Alzheimer's disease. Neurosci Lett 703:53-57 (2019).PubMed: 30885635
• Fattouh N et al. Wolbachia endosymbionts subvert the endoplasmic reticulum to acquire host membranes without triggering ER stress. PLoS Negl Trop Dis 13:e0007218 (2019).PubMed: 30893296

• Sun D et al. Polyubiquitin chain-induced p62 phase separation drives autophagic cargo segregation. Cell Res 28:405-415 (2018).PubMed: 29507397
• Sun J et al. The ubiquitin-specific protease USP8 deubiquitinates and stabilizes Cx43. J Biol Chem 293:8275-8284 (2018).PubMed: 29626091
• Orsi SA et al. Distinct subcellular changes in proteasome activity and linkage-specific protein polyubiquitination in the amygdala during the consolidation and reconsolidation of a fear memory. Neurobiol Learn Mem 157:1-11 (2018).PubMed: 30458285
• Cheng Q et al. Neddylation contributes to CD4+ T cell-mediated protective immunity against blood-stage Plasmodium infection. PLoS Pathog 14:e1007440 (2018).PubMed: 30462731
• Prabakaran T et al. Attenuation of cGAS-STING signaling is mediated by a p62/SQSTM1-dependent autophagy pathway activated by TBK1. EMBO J 37:N/A (2018).PubMed: 29496741
• Lim JA et al. Therapeutic Benefit of Autophagy Modulation in Pompe Disease. Mol Ther 26:1783-1796 (2018).PubMed: 29804932
• Wu Z et al. Ubiquitination of ABCE1 by NOT4 in Response to Mitochondrial Damage Links Co-translational Quality Control to PINK1-Directed Mitophagy. Cell Metab 28:130-144.e7 (2018).PubMed: 29861391
• Shearer RF et al. The E3 ubiquitin ligase UBR5 regulates centriolar satellite stability and primary cilia. Mol Biol Cell 29:1542-1554 (2018).PubMed: 29742019
• Tsuchiya H et al. Ub-ProT reveals global length and composition of protein ubiquitylation in cells. Nat Commun 9:524 (2018). WB .PubMed: 29410401
• Lin YH et al. RavN is a member of a previously unrecognized group of Legionella pneumophila E3 ubiquitin ligases. PLoS Pathog 14:e1006897 (2018). WB .PubMed: 29415051
• Ye B et al. Klf4 glutamylation is required for cell reprogramming and early embryonic development in mice. Nat Commun 9:1261 (2018).PubMed: 29593216
• Mallikarjuna P et al. VHL status regulates transforming growth factor-ß signaling pathways in renal cell carcinoma. Oncotarget 9:16297-16310 (2018).PubMed: 29662646
• Bowen TS et al. Small-molecule inhibition of MuRF1 attenuates skeletal muscle atrophy and dysfunction in cardiac cachexia. J Cachexia Sarcopenia Muscle 8:939-953 (2017). WB ; Mouse .PubMed: 28887874
• Tsuda S et al. Novel mechanism of regulation of the 5-lipoxygenase/leukotriene B4 pathway by high-density lipoprotein in macrophages. Sci Rep 7:12989 (2017).PubMed: 29021582
• Necchi V et al. Natural history of Helicobacter pylori VacA toxin in human gastric epithelium in vivo: vacuoles and beyond. Sci Rep 7:14526 (2017).PubMed: 29109534
• Kitamura H et al. Ubiquitin-Specific Protease 2 Modulates the Lipopolysaccharide-Elicited Expression of Proinflammatory Cytokines in Macrophage-like HL-60 Cells. Mediators Inflamm 2017:6909415 (2017).PubMed: 29138532
• Zhang R et al. OVA12 promotes tumor growth by regulating p53 expression in human cancer cells. Oncotarget 8:52854-52865 (2017).PubMed: 28881777
• Sambri I et al. Lysosomal dysfunction disrupts presynaptic maintenance and restoration of presynaptic function prevents neurodegeneration in lysosomal storage diseases. EMBO Mol Med 9:112-132 (2017). WB ; Mouse .PubMed: 27881461
• Gilmore BL et al. Molecular Analysis of BRCA1 in Human Breast Cancer Cells Under Oxidative Stress. Sci Rep 7:43435 (2017). WB ; Human .PubMed: 28262780
• Li X et al. USP9X regulates centrosome duplication and promotes breast carcinogenesis. Nat Commun 8:14866 (2017). WB .PubMed: 28361952
• Biswas K et al. The E3 Ligase CHIP Mediates p21 Degradation to Maintain Radioresistance. Mol Cancer Res 15:651-659 (2017).PubMed: 28232384
• French ME et al. Mechanism of ubiquitin chain synthesis employed by a HECT domain ubiquitin ligase. J Biol Chem 292:10398-10413 (2017).PubMed: 28461335
• Bromfield EG et al. Proteolytic degradation of heat shock protein A2 occurs in response to oxidative stress in male germ cells of the mouse. Mol Hum Reprod 23:91-105 (2017).PubMed: 27932549
• Guo L et al. NLRC3 promotes host resistance against Pseudomonas aeruginosa-induced keratitis by promoting the degradation of IRAK1. Int J Mol Med 40:898-906 (2017).PubMed: 28731142
• Zhao K et al. Intracellular osteopontin stabilizes TRAF3 to positively regulate innate antiviral response. Sci Rep 6:23771 (2016).PubMed: 27026194
• Qin S et al. XIAP inhibits mature Smac-induced apoptosis by degrading it through ubiquitination in NSCLC. Int J Oncol 49:1289-96 (2016). WB ; Human .PubMed: 27498621
• Fan W et al. TRIM52 inhibits Japanese Encephalitis Virus replication by degrading the viral NS2A. Sci Rep 6:33698 (2016).PubMed: 27667714
• Ramachandran S et al. SYVN1, NEDD8, and FBXO2 Proteins Regulate ?F508 Cystic Fibrosis Transmembrane Conductance Regulator (CFTR) Ubiquitin-mediated Proteasomal Degradation. J Biol Chem 291:25489-25504 (2016).PubMed: 27756846
• Amaya M et al. The ubiquitin proteasome system plays a role in venezuelan equine encephalitis virus infection. PLoS One 10:e0124792 (2015). WB .PubMed: 25927990
• Nardi F et al. Proteasomal modulation of cellular SNAT2 (SLC38A2) abundance and function by unsaturated fatty acid availability. J Biol Chem 290:8173-84 (2015). Human .PubMed: 25653282
• Gilmore BL et al. A Molecular Toolkit to Visualize Native Protein Assemblies in the Context of Human Disease. Sci Rep 5:14440 (2015). IP ; Human .PubMed: 26395823
• Ye J et al. Heat shock protein 70 is associated with replicase complex of Japanese encephalitis virus and positively regulates viral genome replication. PLoS One 8:e75188 (2013). WB ; Human .PubMed: 24086464
• Winton CE et al. A microchip platform for structural oncology applications. NPJ Breast Cancer 2:N/A (0). IP .

Mouse monoclonal anti-ubiquitin (P4D1) antibody for immunoblotting, Cell Signaling, Cat# 3936
Manufacturer website: https://www.cellsignal.com/products/primary-antibodies/ubiquitin-p4d1-mouse-mab/3936
References:
1. Journal: Nat Commun Applications:
Western blotting following immunoprecipitatio...
Reactivity:
Homo sapiens (Human)
Title: Mutually exclusive acetylation and ubiquitylation of the splicing factor SRSF5 control tumor growth.

Author: Yuhan Chen, et. al.
Year: 2018

Rabbit polyclonal Topoisomerase I antibody for immunoblotting, Novus Biologicals,Cat# NBP1-30481
Manufacturer website:https://www.novusbio.com/products/topoisomerase-i-antibody_nbp1-30481
References:
1. 1Kim PY, Tan O, Liu B et al. High TDP43 expression is required for TRIM16-induced inhibition of cancer cell growth and correlated with good prognosis of neuroblastoma and breast cancer patients. Cancer Lett. 2016 Feb 20 [PMID: 26902425] (WB, Human)
Application: WB Species: Human
2. Kim PY, Rahmanto AS, Tan O et al. TRIM16 overexpression induces apoptosis through activation of caspase-2 in cancer cells. Apoptosis 2013 Feb 13 [PMID: 23404198] (WB, Human)
Application: WB Species: Human

Rabbit polyclonal anti-USP3 antibody for immunoblotting, Invitrogen, Cat# PA5-85512, Lot# UF2794327C
This antibody was validated by the manufacturer: https://www.thermofisher.com/antibody/product/USP3-Antibody-Polyclonal/PA5-85512. The antibody was also validated in our western blot experiments both in endogenous and USP3 overexpressing conditions using molecular weight markers.

Mouse anti-ALYREF (11G5) antibody for ChIP and ChIPSeq, ImmunoQuest Ltd. Cat# IQ221
website information: https://www.biocompare.com/9776-Antibodies/1498009-Mouse-monoclonal-AntiALY/
Selected citations:
1. The role of deimination in ATP5b mRNA transport in a transgenic mouse model of multiple sclerosis.EMBO reportsMarch 1, 2012Di Ding, Mabel Enriquez-Algeciras, Kunjan R Dave, Miguel Perez-Pinzon, Sanjoy K Bhattacharya
2. The Cellular DExD/H-Box RNA-Helicases UAP56 and URH49 Exhibit a CRM1-Independent Nucleocytoplasmic Shuttling ActivityPLoS ONEJuly 3, 2011Marco Thomas, Peter Lischka, Regina Müller, Thomas Stamminger
3. Differential expression of THOC1 and ALY mRNP biogenesis/export factors in human cancers.BMC cancerJanuary 1, 2011María S Domínguez-Sánchez, Carmen Sáez, Miguel A Japón, Andrés Aguilera, Rosa Luna
4. Individual influenza A virus mRNAs show differential dependence on cellular NXF1/TAP for their nuclear export.The Journal of general virologyMay 1, 2010Eliot K C Read, Paul Digard
5. Adaptor Aly and co-adaptor Thoc5 function in the Tap-p15-mediated nuclear export of HSP70 mRNA.The EMBO journalMarch 4, 2009Jun Katahira, Hitomi Inoue, Ed Hurt, Yoshihiro Yoneda
6. ATP-dependent recruitment of export factor Aly/REF onto intronless mRNAs by RNA helicase UAP56.Molecular and cellular biologyJanuary 1, 2008Ichiro Taniguchi, Mutsuhito Ohno

Mouse monoclonal anti-β-actin (AC-15) antibody for immunoblotting, Sigma Aldrich, Cat#: A1978
Manufacturer website:https://www.sigmaaldrich.com/catalog/product/sigma/a1978?lang=en&region=AU
selected citations: 1. Gimona, M., et al., Cell Motil. Cytoskel., 27, 108- 116 (1994). 2. North, A.J., et al., J. Cell Sci., 107, 445-455 (1994). 3. North, A.J., et al., J. Cell Sci., 107, 437-444 (1994). 4. Vandekerckhove, J., and Weber, K., Eur. J. Biochem., 90, 451-462 (1978). 5. Drew, J.S., et al., Amer. J. Physiol., 260, C1332- C1340 (1991). 6. Lessard, J.L., Cell Motil. Cytoskel., 10, 349-362 (1988). 7. Sawyer, C., et al., Cancer Res. 63, 1667-1675 (2003). 8. Kalinichenko, V.V., et al., J. Biol. Chem., 277, 12369-12374 (2002). 9. Lacor, P.N., et al., Proc. Natl. Acad. Sci. USA, 97, 3556-3561 (2000). 10. Song, J., et al., J. Histol., 48, 1441-1452 (2000).

Mouse monoclonal anti-GAPDH (G-9) antibody for immunoblotting, Santa Cruz Biotechnology, Cat# sc-365062, Lot#C2119
Manufacturer website: https://www.novusbio.com/products/topoisomerase-i-antibody_nbp1-30481
selected citations: 1. Tang, L., et al. 2006. The principal urinary metabolites of dietary isothiocyanates, N-acetylcysteine conjugates, elicit the same anti-proliferative response as their parent compounds in human bladder cancer cells. Anticancer Drugs 17: 297-305. 2. Tang, L., et al. 2006. Potent activation of mitochondria-mediated apoptosis and arrest in S and M phases of cancer cells by a broccoli sprout extract. Mol. Cancer Ther. 5: 935-944. 3. Saba, J., et al. 2019. Melanocortin 4 receptor activation protects striatal neurons and glial cells from 3-nitropropionic acid toxicity. Mol. Cell. Neurosci. 94: 41-51. 4. Ito, S., et al. 2019. Knockdown of orphan transporter SLC22A18 impairs lipid metabolism and increases invasiveness of HepG2 cells. Pharm. Res. 36: 39. 5. Xiong, R., et al. 2019. Disease-related responses induced by cadmium in an in vitro human airway tissue model. Toxicol. Lett. 303: 16-27. 6. Hu, W., et al. 2019. Vitamin D3 activates the autolysosomal degradation function against Helicobacter pylori through the PDIA3 receptor in gastric epithelial cells. Autophagy. E-published. 7. Wang, W.B., et al. 2019. CTGF regulates cyclic stretch-induced vascular smooth muscle cell proliferation via microRNA-19b-3p. Exp. Cell Res. E-published. 8. Wu, Z., et al. 2019. WT1-interacting protein inhibits cell proliferation and tumorigenicity in non-small cell lung cancer via the AKT/FOXO1 axis. Mol. Oncol. E-published.

Rabbit control IgG antibody for immunoprecipitation, Cell Signaling, Cat#2729
Manufacturer website: https://www.scbt.com/p/normal-mouse-igg
Citations:
Journal: Mucosal Immunol Applications:
Immunoprecipitation (IP)
Reactivity:
Homo sapiens (Human)
Title: Alpha-hemolysin of uropathogenic Escherichia coli induces GM-CSF-mediated acute kidney injury.
Author: Changying Wang, et. al.
Year: 2020

Journal: Nat Commun Applications:
Immunoprecipitation (IP)
Reactivity:
Homo sapiens (Human)
Title: ARID1A and PI3-kinase pathway mutations in the endometrium drive epithelial transdifferentiation and collective invasion...
Author: Mike R Wilson, et. al.

Year: 2019

Journal: Nat Commun Applications:
Immunoprecipitation (IP)
Reactivity:
Homo sapiens (Human)
Title: A MST1-FOXO1 cascade establishes endothelial tip cell polarity and facilitates sprouting angiogenesis.
Author: Yoo Hyung Kim, et. al.
Year: 2019

Journal: Nat Commun Applications:
Immunoprecipitation (IP)
Reactivity:
Homo sapiens (Human)
Title: A non-canonical BRD9-containing BAF chromatin remodeling complex regulates naive pluripotency in mouse embryonic stem ce...
Author: Jovylyn Gatchalian, et. al.
Year: 2018

Journal: Cell Death Dis Applications:
Immunoprecipitation (IP)
Reactivity:
Homo sapiens (Human)
Title: Periostin secreted by cancer-associated fibroblasts promotes cancer stemness in head and neck cancer by activating prote...
Author: Binbin Yu, et. al.
Year: 2018

Journal: Nat Commun Applications:
Immunoprecipitation (IP)
Reactivity:
Homo sapiens (Human)
Title: CFP1 coordinates histone H3 lysine-4 trimethylation and meiotic cell cycle progression in mouse oocytes.
Author: Qian-Qian Sha, et. al.
Year: 2018

Journal: Dev Cell Applications:
Immunoprecipitation (IP)
Reactivity:
Homo sapiens (Human)
Title: The RAB11A-Positive Compartment Is a Primary Platform for Autophagosome Assembly Mediated by WIPI2 Recognition of PI3P-R...
Author: Claudia Puri, et. al.
Year: 2018

Journal: Sci Rep Applications:
Immunoprecipitation (IP)
Reactivity:
Homo sapiens (Human)
Title: Collagen Type 1 Accelerates Healing of Ruptured Fetal Membranes.
Author: Haruta Mogami, et. al.
Year: 2018

Journal: Genes Dev Applications:
Immunoprecipitation (IP)
Reactivity:
Homo sapiens (Human), Mus musculus (House mouse)
Title: Foxp1 regulation of neonatal vocalizations via cortical development.
Author: Noriyoshi Usui, et. al.
Year: 2017

Journal: Mol Cell Applications:
Chromatin immunoprecipitation (ChIP), Immunop...
Reactivity:
Homo sapiens (Human)
Title: Bromodomain Protein BRD4 Is a Transcriptional Repressor of Autophagy and Lysosomal Function.
Author: Jun-Ichi Sakamaki, et. al.
Year: 2017

Journal: Nucleic Acids Res Applications:
Immunoprecipitation (IP)
Reactivity:
Homo sapiens (Human)
Title: S6K2-mediated regulation of TRBP as a determinant of miRNA expression in human primary lymphatic endothelial cells.
Author: Matthew J Warner, et. al.

Year: 2016

Journal: J Biol Chem Applications:
Immunoprecipitation (IP)
Reactivity:
Homo sapiens (Human)
Title: HIV-1 Vpr Protein Induces Proteasomal Degradation of Chromatin-associated Class I HDACs to Overcome Latent Infection of ...
Author: Bizhan Romani, et. al.
Year: 2016

Journal: Nucleic Acids Res Applications:
Immunoprecipitation (IP)
Reactivity:
Homo sapiens (Human)
Title: Nuclear pyruvate kinase M2 complex serves as a transcriptional coactivator of arylhydrocarbon receptor.
Author: Shun Matsuda, et. al.
Year: 2016

Journal: EMBO J Applications:
Immunoprecipitation (IP)
Reactivity:
Homo sapiens (Human), Mus musculus (House mouse)
Title: The Mediator subunit MED23 couples H2B mono-ubiquitination to transcriptional control and cell fate determination.

Author: Xiao Yao, et. al.
Year: 2015

Journal: Nature Applications:
Immunoprecipitation (IP)
Reactivity:
Homo sapiens (Human)
Title: CMT2D neuropathy is linked to the neomorphic binding activity of glycyl-tRNA synthetase.
Author: Weiwei He, et. al.
Year: 2015

Journal: Elife Applications:
Immunoprecipitation (IP)
Reactivity:
Homo sapiens (Human)
Title: Ferritinophagy via NCOA4 is required for erythropoiesis and is regulated by iron dependent HERC2-mediated proteolysis.
Author: Joseph D Mancias, et. al.
Year: 2015

Journal: Nat Commun Applications:
Immunoprecipitation (IP)
Reactivity:
Homo sapiens (Human)
Title: A caveolin-dependent and PI3K/AKT-independent role of PTEN in β-catenin transcriptional activity.
Author: Alejandro Conde-Perez, et. al.
Year: 2015

Journal: BMC Cancer Applications:
Chromatin immunoprecipitation (ChIP), Immunop...
Reactivity:
Homo sapiens (Human)
Title: Wilms' tumor gene 1 regulates p63 and promotes cell proliferation in squamous cell carcinoma of the head and neck
Author: Xingru Li, et. al.
Year: 2015

Journal: Nat Commun Applications:
Chromatin immunoprecipitation (ChIP), Immunop...
Reactivity:
Homo sapiens (Human)
Title: Transcription factors FOXG1 and Groucho/TLE promote glioblastoma growth.
Author: Federica Verginelli, et. al.
Year: 2013

Journal: Nat Biotechnol Applications:
Immunoprecipitation (IP)
Reactivity:
Homo sapiens (Human)
Title: Protein interaction discovery using parallel analysis of translated ORFs (PLATO).
Author: Jian Zhu, et. al.
Year: 2013

Journal: J Biol Chem Applications:
Immunoprecipitation (IP)
Reactivity:
Homo sapiens (Human)
Title: Annexin 2 regulates endothelial morphogenesis by controlling AKT activation and junctional integrity.
Author: Shih-Chi Su, et. al.
Year: 2010

Mouse control IgG antibody for immunoprecipitation, Santa Cruz Biotechnology, Cat# sc-2025
Manufacturer website:https://www.scbt.com/p/normal-mouse-igg
selected citations: 1. Tan, M., et al. 2002. Phosphorylation on tyrosine-15 of p34(Cdc2) by ErbB2 inhibits p34(Cdc2) activation and is involved in resistance to taxol-induced apoptosis. Mol. Cell 9: 993-1004. 2. Acevedo-Duncan, M., et al. 2002. Human glioma PKC-iota and PKC-βII phosphorylate cyclin-dependent kinase activating kinase during the cell cycle. Cell Prolif. 35: 23-36. 3. Xie, R., et al. 2011. Microtubule-associated protein 1S (MAP1S) bridges autophagic components with microtubules and mitochondria to affect autophagosomal biogenesis and degradation. J. Biol. Chem. 286: 10367-10377. 4. Bernatik, O., et al. 2011. Sequential activation and inactivation of dishevelled in the Wnt/β-catenin pathway by casein kinases. J. Biol. Chem. 286: 10396-10410. 5. Lenasi, T., et al. 2011. Cap-binding protein complex links pre-mRNA capping to transcription elongation and alternative splicing through positive transcription elongation factor β (P-TEFβ). J. Biol. Chem. 286: 22758-22768. 6. Gizard, F., et al. 2011. Telomerase activation in atherosclerosis and induction of telomerase reverse transcriptase expression by inflammatory stimuli in macrophages. Arterioscler. Thromb. Vasc. Biol. 31: 245-252. 7. Euskirchen, G.M., et al. 2011. Diverse roles and interactions of the SWISNF chromatin remodeling complex revealed using global approaches. PLoS Genet. 7: e1002008. 8. Li, B., et al. 2011. Increased hedgehog signaling in postnatal kidney results in aberrant activation of nephron developmental programs. Hum. Mol. Genet. 20: 4155-4166. 9. Liu, J., et al. 2011. Hyperglycemia-induced cerebral hematoma expansion is mediated by plasma kallikrein. Nat. Med. 17: 206-210. 10.Calaf, G.M., et al. 2011. Protective role of curcumin in oxidative stress of breast cells. Oncol. Rep. 26: 1029-1035. 11.Song, Y., et al. 2011. Ligand-dependent corepressor acts as a novel corepressor of thyroid hormone receptor and represses hepatic lipogenesis in mice. J. Hepatol. 56: 248-254. 12.Taura, M., et al. 2011. MEF/ELF4 transactivation by E2F1 is inhibited by p53. Nucleic Acids Res. 39: 76-88. 13.Chen, S.T., et al. 2012. Recombinant MPT83 derived from Mycobacterium tuberculosis induces cytokine production and upregulates the function of mouse macrophages through TLR2. J. Immunol. 188: 668-677. 14.Reid, H.M., et al. 2012. Interaction of the human prostacyclin receptor and the NHERF4 family member intestinal and kidney enriched PDZ protein (IKEPP). Biochim. Biophys. Acta 1823: 1998-2012. 15.Zanin-Zhorov, A., et al. 2012. Scaffold protein Disc large homolog 1 is required for T-cell receptor-induced activation of regulatory T-cell function. Proc. Natl. Acad. Sci. USA 109: 1625-1630. 16.Kim, Y.C., et al. 2012. Angiotensin II regulates activation of Bim via Rb/E2F1 during apoptosis: involvement of interaction between AMPKβ1/2 and Cdk4. Am. J. Physiol. Lung Cell Mol. Physiol. 303: L228-L238.

Mouse anti-BrdU for BrdU assay, part of BrdU proliferation kit by Roche, Cat# 11647229001 and
Anti-mouse IgG-Alexa-Flour-594 for BrdU assay, part of BrdU proliferation kit by Roche, Cat# 11647229001
Manufacturer website:https://www.sigmaaldrich.com/catalog/product/roche/11647229001?lang=en&region=AU
References:
1 Gratzner, H. G. (1982) Science 218, 474-475. 2 Allison, L. et al. (1985) Cytometry 6, 584-590. 3 Vanderlaan, M. & Thomas, C. B. (1985) Cytometry 6, 501-505. 4 Gonchoroff, N. J. et al. (1985) Cytometry 6, 506-512. 5 Harms, G. et al. (1986) Histochemistry 85, 139-143. 6 Schutte, B. et al. (1987) J. Histochem. Cytochem. 35, 371-374. 7 Porstmann, T., Ternyck, T. & Avrameas, S. (1985) J. Immunol. Methods 82, 169-179. 8 Magaud, J.-P., Sargent, I. & Mason, D. Y. (1988) J. Immunol. Methods 106, 95-100. 9 Huong, P. L. T. et al. (1991) J. Immunol. Methods 140, 243-248. 10 Muir, D., Varon, S. & Manthorpe, M. (1990) Anal. Biochem. 185, 377-382. 11 Heil, J. & Reifferscheid, G. (1992) Carcinogenesis 13, 2389-2394. 12 Rußmann, E. et al. (1993) Colloquium Roche Molecular Biochemicals 4, 1-4. 13 Current Protocols in Immunology 1, chapter 7.10. 1 (Coligan, J. E. et al., eds.) John -Wiley & Sons, New York

Eukaryotic cell lines

Policy information about cell lines

Cell line source(s)	Neuroblastoma cell line SHEPMYCN3 was kindly provided by Professor Jason Shohet (Texas Children's Cancer Center, Houston, TX, USA). Neuroblastoma cell lines, SK-N-BE(2)C, SH-EP and SH-SY5Y cells were provided by Barbara Spengler (Fordham University, New York, NY). Neuroblastoma cell lines, IMR-32 and SK-N-FI as well as HEK293T cells were obtained from the American Type Culture Collection (Manassas, VA). The Lenti-X™ 293T viral packaging cell line was purchased from Scientifix (South Yarra, Victoria, Australia). Neuroblastoma cell lines, Kelly, CHP-134, SK-N-DZ and SK-N-AS cells were obtained from the European Collection of Cell Cultures through Sigma (Sigma, Sydney, Australia). Neuroblastoma cell line NBLS was kindly provided by Prof. Susan L. Cohn (Northwestern University, Chicago, IL, USA). Neuroblastoma cell line LAN-1 was kindly provided by Dr. John Maris (Children's Hospital of Philadelphia, Philadelphia, USA). MRC-5 and WI-38 normal human fibroblasts were purchased from ATCC (Manassas, VA, USA). Stable neuroblastoma cells for USP3 overexpression or ALYREF knock-down were derived from SK-N-BE(2)C and Kelly cells.
Authentication	The identity of cell lines was verified in 2010, 2014, 2015, 2016 and 2017 by short tandem repeat profiling conducted at the Garvan Institute of Medical Research or Cellbank Australia.
Mycoplasma contamination	All cell lines were confirmed to be mycoplasma free.
Commonly misidentified lines (See ICLAC register)	No commonly misidentified cell lines were used.

Animals and other organisms

Policy information about studies involving animals; ARRIVE guidelines recommended for reporting animal research

Laboratory animals	Female Balb/c nude mice aged 5 to 6 weeks were obtained from Australian BioResources Mossvale, NSW, Australia. All mice were culled when tumor volume reached 1 cm3. Maximum holding time was 12 weeks. Mice were held under PC2 conditions in individually ventilated caging with a maximum of 6 or a minimum of 2 mice per cage. Dimensions for mice cages: 369 X 156 X 132 mm, with a floor area of 440 cm2. The mice were maintained in a protected and controlled environment. The animal facility was barrier protected with the air HEPA filtered and the room maintained at positive pressure and at a temperature 22 +/- 1 degree Celsius. Sterile feed and water were provided ad libitum. The light in the animal facility was on a 12h cycle. All experimental work was conducted in biosafety or cytotoxic cabinets to further protect the animals from microbiological threat.
Wild animals	No wild animals were used in this study.
Field-collected samples	No field-collected samples were used in this study.
Ethics oversight	Animal experiments were approved by the Animal Care and Ethics Committee of University of New South Wales (ACEC#18/113B), Australia, and the animals were cared for in agreement with institutional guidelines.

Note that full information on the approval of the study protocol must also be provided in the manuscript.

ChIP-seq

Data deposition

☒ Confirm that both raw and final processed data have been deposited in a public database such as GEO.

☒ Confirm that you have deposited or provided access to graph files (e.g. BED files) for the called peaks.

Data access links<br>*May remain private before publication.*	The ALYREF ChIP sequencing data has been deposited at the Gene Expression Omnibus website with series number of GSE150303.<br>https://www.ncbi.nlm.nih.gov/geo/query/acc.cgi?acc=GSE150303
Files in database submission	Both raw and processed data for SK-N-BE(2)-C genomic input and SK-N-BE(2)-C ALYREF antibody ChIP have been deposited under GSE150303.<br><br>Processed data files:<br>BE2C_ALYREF_peaks_annotated.txt<br>BE2C_ALYREF_FE.bw<br><br>Raw data files:<br>BE2C-ALY_FKDL190732348-1a-2_1.fq.gz<br>BE2C-ALY_FKDL190732348-1a-2_2.fq.gz<br>BE2C-input_FKDL190732347-1a-4_1.fq.gz<br>BE2C-input_FKDL190732347-1a-4_2.fq.gz
Genome browser session<br>(e.g. UCSC)	https://genome.ucsc.edu/cgi-bin/hgTracks?db=hg38&lastVirtModeType=default&lastVirtModeExtraState=&virtModeType=default&virtMode=0&nonVirtPosition=&position=chr15%3A63477030%2D63567070&hgsid=834951129_kXSqZcMN0NpFu3n3gX5P3xbAASiC

Methodology

Replicates	Experiment was performed once and was based on previous experience with experimental setup. The sample size is acceptable in the field.
Sequencing depth	Samples were analysed using Illumina Hiseq X-Ten pair end sequencing (150 bp). BE2C-ALY samples were sequenced at a depth of 5.62x10^7 paired end reads and BE2C-input was sequenced at a depth of 3.08x10^7 paired end reads.
Antibodies	Mouse anti-ALYREF (11G5) antibody for ChIP and ChIPSeq, ImmunoQuest Ltd. Cat# IQ221<br>Mouse control IgG antibody for immunoprecipitation, Santa Cruz Biotechnology, Cat# sc-2025
Peak calling parameters	Peaks were called for using MACS2 (v2.1.1) in paired end mode with an FDR q-value threshold of 0.05 with parameters (-B -q 0.05 -f BAMPE --SPMR -g hs).
Data quality	To ensure data quality we initially quality trimmed sequencing reads and kept only those reads which had valid pairs. We also removed reads aligning to ENCODE blacklisted regions to ensure we would not call false-positive peaks in these regions. For downstream analyses, we only considered those peaks which could be confidently assigned to a gene/promoter. There were a total of 381 peaks at an FDR of 5% and > 5 fold-enrichment.
Software	Reads from fastq files were first quality trimmed using TrimGalore (v0.4.5) using parameters (--phred33 --fastqc --illumina --paired --quality 20 --length 20). Reads were aligned to the human reference genome (GRCh38) using bowtie2 (v2.1.0) using parameters (--local --no-unal -X 2000), resulting sam files were converted to bam files and sorted using samtools (v1.9).

Reads aligned to ENCODE blacklisted regions were removed using bedtools (v2.27.1). Peaks were then called using MACS2 (v2.1.1) in paired end mode for with an FDR q-value threshold of 0.05 with parameters (-B -q 0.05 -f BAMPE --SPMR -g hs). Fold enrichment tracks were also generated using MACS2 (v2.1.1) and converted to the bigwig format using bedtools (v2.27.1). HOMER (v4.10.3) was used to annotate peaks (promoters were considered to be -1000bp/+100bp from the transcription start site (TSS)) and then to perform de novo/known motif discovery on ALYREF peak regions using parameters (-size 200 -p 8). GRCh38 annotated peak text file produced from MACS2 (v2.1.1) and then annotated using HOMER (v4.10.3) bigWig file generated by converting MACS2 (v2.1.1) fold enrichment bedgraph file using bedtools (v2.27.1), scores represent fold enrichment values of ALYREF compared to genomic input.
